# Defining the regulon of genes controlled by σ^E^, a key regulator of the cell envelope stress response in *Streptomyces coelicolor*

**DOI:** 10.1111/mmi.14250

**Published:** 2019-04-29

**Authors:** Ngat T. Tran, Xiaoluo Huang, Hee‐Jeon Hong, Matthew J. Bush, Govind Chandra, Daniela Pinto, Maureen J. Bibb, Matthew I. Hutchings, Thorsten Mascher, Mark J. Buttner

**Affiliations:** ^1^ Department of Molecular Microbiology John Innes Centre Norwich Research Park Norwich NR4 7UH UK; ^2^ Department Biology I Ludwig‐Maximilians‐Universität München Großhaderner Str. 2‐4 Planegg‐Martinsried 82152 Germany; ^3^ School of Biological Sciences University of East Anglia Norwich Research Park Norwich NR4 7TJ UK; ^4^ Present address: Department of Biological and Medical Sciences Oxford Brookes University Oxford OX3 0BP UK; ^5^ Present address: Institut für Mikrobiologie Technische Universität Dresden Dresden 01062 Germany

## Abstract

The extracytoplasmic function (ECF) σ factor, σ^E^, is a key regulator of the cell envelope stress response in *Streptomyces coelicolor*. Although its role in maintaining cell wall integrity has been known for over a decade, a comprehensive analysis of the genes under its control has not been undertaken. Here, using a combination of chromatin immunoprecipitation‐sequencing (ChIP‐seq), microarray transcriptional profiling and bioinformatic analysis, we attempt to define the σ^E^ regulon. Approximately half of the genes identified encode proteins implicated in cell envelope function. Seventeen novel targets were validated by S1 nuclease mapping or *in vitro* transcription, establishing a σ^E^‐binding consensus. Subsequently, we used bioinformatic analysis to look for conservation of the σ^E^ target promoters identified in *S. coelicolor* across 19 *Streptomyces* species. Key proteins under σ^E^ control across the genus include the actin homolog MreB, three penicillin‐binding proteins, two L,D‐transpeptidases, a LytR‐CpsA‐Psr‐family protein predicted to be involved in cell wall teichoic acid deposition and a predicted MprF protein, which adds lysyl groups to phosphatidylglycerol to neutralize membrane surface charge. Taken together, these analyses provide biological insight into the σ^E^‐mediated cell envelope stress response in the genus *Streptomyces*.

## Introduction

The bacterial cell envelope, made up of the cell wall and cell membranes, is critical in counteracting the high intracellular osmotic pressure to maintain cell shape (Silhavy *et al.*, 2010). It also provides an essential defensive barrier against various environmental stress agents. The cell envelope facilitates the ability of the cell to monitor the external environment and modulate cell behaviour in response (Jordan *et al.*, 2008). Numerous antibiotics target the bacterial cell envelope. For example, penicillin and other β‐lactams mimic the D‐alanyl‐D‐alanine (D‐ala‐D‐ala) terminus of the pentapeptide side chain of peptidoglycan and thus block the activity of penicillin‐binding proteins (PBPs) in the elongation and cross‐linking of peptidoglycan precursors. Furthermore, vancomycin and other glycopeptide antibiotics bind to the D‐ala‐D‐ala terminus and thereby inhibit peptidoglycan cross‐linking (Kahne *et al.*, 2005).

**Figure 1:**
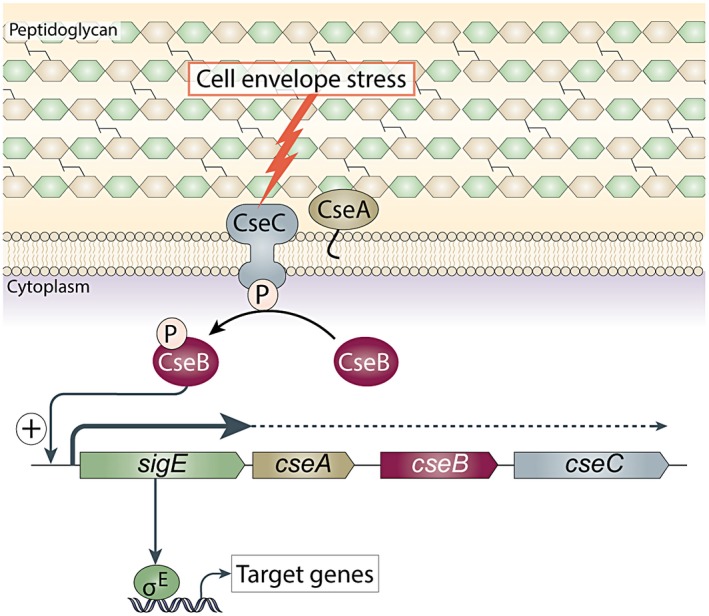
Model for the σ^E^ cell envelope stress response. Expression of the gene encoding σ^E^ (*sigE*) is regulated at the level of transcription by the CseB/CseC two‐component signal transduction system. In response to signals originating in the cell envelope when it is under stress, the sensor kinase, CseC, becomes autophosphorylated and transfers this phosphate to the response regulator, CseB. Phospho‐CseB activates the promoter of the *sigE* operon, and σ^E^ is recruited by core RNA polymerase to transcribe its regulon. Note that >90% transcription from the *sigE* promoter terminates just downstream of *sigE* and that the promoter of the *sigE* gene itself is not a σ^E^ target. CseA is a lipoprotein localised to the extracytoplasmic face of the cell membrane and loss of the CseA results in upregulation of the *sigE* promoter.

Bacteria employ two major types of signalling system to sense and respond to environmental stresses: two‐component systems and extracytoplasmic function (ECF) σ factors (Raivio, 2005; Jordan *et al.*, 2008; Mitrophanov and Groisman, 2008; Capra and Laub, 2012). These two systems are functionally analogous in that they generally consist of a membrane protein (a sensor kinase or an anti‐σ factor) that acts as a stress sensor and a transcription factor (a response regulator or a σ factor) that modulates gene expression in response. In the case of two‐component systems, the inducing signal leads to the autophosphorylation of a membrane‐bound sensor kinase. As a result, the kinase phosphorylates its cognate response regulator, which then activates transcription of the genes involved in the cellular response (Mitrophanov and Groisman, 2008; Capra and Laub, 2012). Similarly, ECF σ factors typically control the cellular stress response via an interaction with a cognate anti‐σ factor, which is usually a transmembrane protein (Mascher, 2013). In the absence of the signal, the anti‐σ factor sequesters its cognate ECF σ factor to the membrane, inhibiting its activity. The inducing signal inactivates the anti‐σ factor, either by causing a conformational change in the protein, or by proteolysis (Mascher, 2013). In either case, the result is the release of the ECF σ factor, which is then able to direct RNA polymerase (RNAP) to its target promoters and elicit a specific transcriptional response. *Streptomyces coelicolor* σ^E^, the subject of this work, is unusual in that it is not regulated by an anti‐σ. Instead, *S. coelicolor* σ^E^ activity is controlled at the level of transcription of its structural gene (*sigE*) by a two‐component system, CseBC (see below) (Fig. 1).

The roles of the two‐component system CpxAR and the ECF σ factor σ^E^ in the cell envelope stress response of *Escherichia coli* have been well established (Ruiz and Silhavy, 2005; Guest and Raivio, 2016). The CpxAR system is induced by a variety of cell envelope stresses including alkaline pH, increased osmolality, overexpression of the outer membrane protein NplE, altered membrane composition, and the accumulation of pilus subunits or misfolded MalE aggregates (Guest and Raivio, 2016). Activation of CpxAR results in the elevated expression of target genes that are involved in envelope protein folding and degradation, such as the periplasmic protease DegP, the periplasmic disulfide oxidoreductase DsbA and the foldase chaperone PpiA (Guest and Raivio, 2016). The *E. coli* ECF σ factor σ^E^ mainly responds to stresses that affect the folding of outer membrane proteins (OMPs) such as heat shock (Rouviere *et al.*, 1995). In line with this, mutations in the OMP folding chaperone also induce the σ^E^ stress response (Missiakas *et al.*, 1996). The σ^E^ regulon includes a variety of genes involved in OMP folding (Dartigalongue *et al.*, 2001; Rhodius *et al.*, 2005) and several small RNAs that downregulate OMP expression, thereby reducing the flow of OMPs to the cell envelope (Johansen *et al.*, 2006; Thompson *et al.*, 2007; Udekwu and Wagner, 2007).

In *Bacillus subtilis*, four two‐component systems (LiaRS, BceRS, YvcPQ, YxdJK) and at least four of its seven ECF σ factors, σ^M^, σ^X^, σ^V^, σ^W^, have roles in the response to cell envelope stress (Jordan *et al.*, 2008; Hastie *et al.*, 2014; 2016; Lewerke *et al.*, 2018). For example, BceRS is strongly induced by bacitricin and is involved in bacitracin detoxification (Mascher *et al.*, 2003). σ^M^ is activated by a wide variety of sources of envelope stress such as vancomycin, bacitracin, phosphomycin and cationic antimicrobial peptides (Mascher *et al.*, 2003; Thackray and Moir, 2003; Kingston *et al.*, 2013). Much effort has also been made to define the regulatory networks linked to these signalling systems. σ^M^ contributes to the transcription of genes whose functions are related to transcriptional control, cell wall biosynthesis, cell shape determination, cell division, DNA monitoring and repair, and detoxification (Eiamphungporn and Helmann, 2008). Approximately 57 genes (30 operons) are direct targets of σ^M^ under antibiotic stress conditions, including several targets that also belong to the σ^X^ and/or σ^W^ regulons (Eiamphungporn and Helmann, 2008).

*Streptomyces coelicolor* is a soil dwelling, saprophytic actinobacterium with a complex differentiating life cycle involving filamentous growth and sporulation (Flärdh and Buttner, 2009), and it is a well‐established model organism in which to study signal transduction in the *Streptomyces* genus (Hutchings *et al.*, 2004)*. S. coelicolor* encodes 67 paired two‐component systems (Hutchings *et al.*, 2004), and 51 ECF σ factors (collected from Mist2 database, http://mistdb.com/) (Ulrich and Zhulin, 2010). Of these, only the two‐component systems VanRS and CseBC and the ECF σ factor σ^E^ have so far been shown to play a role in the cell envelope stress response. VanRS controls the expression of an inducible vancomycin resistance cluster of seven genes (*vanSRJKHAX*) (Hong *et al.*, 2004; Hutchings *et al.*, 2006a), and vancomycin activates the VanRS two‐component system directly by binding to the sensor kinase VanS (Koteva *et al.*, 2010). Expression of the *vanHAX* genes reprograms cell wall biosynthesis such that the stem pentapeptide of peptidoglycan precursors terminate in D‐alanyl‐D‐lactate (D‐Ala‐D‐Lac), rather than in D‐Ala‐D‐Ala (Hong *et al.*, 2005). The affinity of vancomycin for precursors terminating in D‐Ala‐D‐Lac is ~1000‐fold lower than for precursors terminating D‐Ala‐D‐Ala (Bugg *et al.*, 1991), thus rendering *S. coelicolor* resistant. VanRS responds specifically to glycopeptide antibiotics like vancomycin, ristocetin, chloroeremomycin and A47934, but not to other cell envelope‐specific antibiotics with different modes of action like the phosphoglycolipid moenomycin A, the peptide bacitracin and the cyclic depsipeptide ramoplanin (Hong *et al.*, 2004; Hutchings *et al.*, 2006a). In contrast, the expression of *S. coelicolor* σ^E^ is induced by a diverse range of antibiotics that target the cell wall, including penicillins, cephalosporins, glycopeptides, moenomycin A, bacitracin and ramoplanin (Hong *et al.*, 2002). A *sigE* mutant shows a 50‐fold increase in sensitivity to the cell wall hydrolytic enzyme lysozyme and a subtle alteration in its cell wall muropeptide profile (Paget *et al.*, 1999a). In addition, *sigE* mutants require high levels of magnesium for normal growth and development and overproduce actinorhodin and form crenelated colonies in its absence (Paget *et al.*, 1999a). It therefore seems likely that Mg^2+^ stabilizes the defect in the cell envelope of *sigE* mutants, thereby suppressing the phenotype. High levels of magnesium are known to suppress a wide range of cell envelope defects in bacteria (Formstone and Errington, 2005). Thus, while the VanRS system is dedicated to glycopeptide resistance, σ^E^ seems to play a much more general role in the response of *S. coelicolor* to cell envelope stress.

The initial characterization of the *S. coelicolor sigE* gene led directly to the discovery of the ECF subfamily of σ factors 25 years ago (Lonetto *et al.*, 1994). The *sigE* gene is located in a four‐gene operon, *sigE cseA cseB cseC*, with *cseA* encoding a lipoprotein, *cseB* encoding a response regulator and *cseC* encoding a membrane‐anchored sensor kinase (Fig. 1). Approximately 90% of transcription terminates directly downstream of the *sigE* gene and transcription of *sigE* is completely dependent on the two‐component system, CseBC (Paget *et al.*, 1999b; Hong *et al.*, 2002). By analogy with other two‐component systems, it seems likely that in response to cell envelope stress, the sensor kinase CseC autophosphorylates before phosphorylating its cognate response regulator, CseB, that in turn directs the transcription of *sigE* (Paget *et al.*, 1999b; Hong *et al.*, 2002) (Fig. 1). The *sigE* promoter seems to be the sole target of CseB since the *sigE* mutant and *cseB* mutant show the same phenotype and constitutive expression of σ^E^ complements the lysozyme sensitivity of *S. coelicolor* lacking CseB (Paget *et al.*, 1999b). The function of the lipoprotein CseA remains unknown, but deletion of *cseA* results in upregulation of the *sigE* promoter, raising the speculative possibility that CseA might modulate the activity of the signal transduction system by interacting with the extracytoplasmic domain of the CseC sensor kinase (Hutchings *et al.*, 2006b). The absence of an anti‐σ and the requirement of a two‐component system for transcription of *sigE* sets this system apart from other well‐characterized ECF σ factor regulatory mechanisms. Thus, despite having the same name, *S. coelicolor* σ^E^ is distinct from both *E. coli* σ^E^ and *Mycobacterium tuberculosis* σ^E^, which instead employ an anti‐σ factor to control ECF σ factor activity (Sineva *et al.*, 2017).

Despite the critical role of σ^E^ in modulating the cell envelope stress response in *S. coelicolor*, only two *in vivo* targets have so far been described: the *hrdD* gene, encoding another σ factor (Paget *et al.*, 1999a), the function of which is poorly understood (Buttner *et al.*, 1990; Strakova *et al.*, 2014), and the 12‐gene *cwg* operon, predicted to be involved in the biosynthesis of a cell wall glycan (Hong *et al.*, 2002). To gain a broader picture of the physiological function of σ^E^ in the cell envelope stress response in *Streptomyces*, here we use a combination of ChIP‐seq, microarray transcriptional profiling and bioinformatic analysis to define the regulon of genes under σ^E^ control. Over 50 targets were found to be directly involved in cell envelope‐related functions and many other targets are implicated in signal transduction systems. Finally, we used bioinformatic analysis to identify *S. coelicolor* σ^E^ target promoters that are conserved across the *Streptomyces* genus. The σ^E^‐directed cell envelope stress response characterized here is likely to be specific to the streptomycetes, because the *sigE*‐*cseABC* operon appears to be absent outside this genus (http://www.microbesonline.org/ [Dehal *et al.*, 2010]).

## Results and discussion

### Identification of the σ^E^ regulon

**Figure 2:**
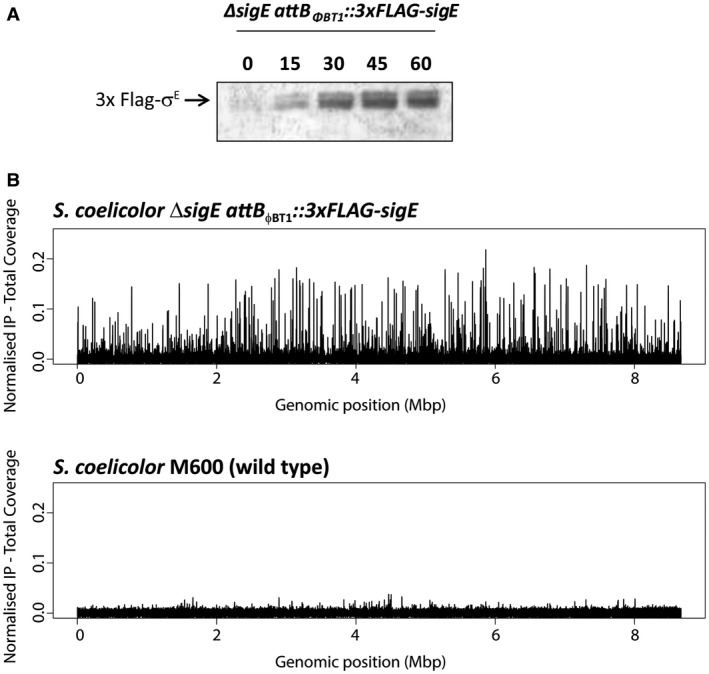
A. Western blot analysis of *S. coelicolor* Δ*sigE attB_ΦBT1_::3 × FLAG‐sigE* grown in NMMP liquid cultures and sampled after 0, 15, 30, 45 and 60 minutes treatment with 10 µg/ml vancomycin. Total protein (10 µg) was loaded per lane and 3 × FLAG‐σ^E^ was detected using anti‐σ^E^ polyclonal antibody. B. Chromosome‐wide distribution of σ^E^‐binding sites in *S. coelicolor* identified by ChIP‐seq analysis. ChIP‐seq was conducted using M2 anti‐FLAG antibody on the Δ*sigE attB_ΦBT1_::3 × FLAG‐sigE* strain after 30 minutes treatment with 10 µg/ml vancomycin. The wild‐type strain (expressing non‐tagged σ^E^ from the native locus) analysed under the same conditions was used as a negative control.

To define the genes under direct control of σ^E^, we used chromatin immunoprecipitation coupled with high‐throughput sequencing (ChIP‐seq). To do this, we first constructed a strain of *S. coelicolor* that lacked *sigE* at its native locus but expressed an N‐terminally triple‐FLAG‐tagged version of σ^E^ from the ΦBT1 integration site. As shown in Fig. S1, expression of 3 × FLAG‐σ^E^ *in trans*, under control of its native promoter, restores the resistance of *S. coelicolor* to lysozyme to wild‐type levels. Furthermore, Western blot analysis showed that vancomycin induced expression of 3 × FLAG‐σ^E^ (Fig. 2A) in the same way that it induces expression of native σ^E^ in wild‐type *S. coelicolor* (Hong *et al.*, 2002).

ChIP‐seq was conducted using M2 anti‐FLAG antibody after 30 minutes of treatment with vancomycin to induce 3 × FLAG‐σ^E^ expression. The congenic wild‐type *S. coelicolor* strain M600 was used as a negative control to eliminate any false signals that might arise from cross‐reaction of the anti‐FLAG antibody with other DNA‐binding proteins. In addition, total (non‐immunoprecipitated) input DNA was also subjected to sequencing. This additional control enables non‐uniform shearing of the chromosome to be taken into account (Teytelman *et al.*, 2009). Using P < 10^−4^ as the threshold for significance, a total of ~200 peaks were detected in the FLAG‐tagged SigE strain (Fig. 2B and Table S1). Notably, only a few small peaks were detected in the wild‐type M600 control strain expressing the non‐tagged version of σ^E^ (Fig. 2B). Next, we looked for candidate σ^E^ target promoter sequences for each ChIP‐seq target, based on the conservation of AAC and TC, respectively, in the −35 and −10 regions of the two previously characterised σ^E^ target promoters, *hrdD* and *cwg* (Paget *et al.*, 1999a; Hong *et al.*, 2002). Restricting our search to within 400 bp of the start codon of the downstream gene, we identified 91 putative σ^E^ target promoters through this route (Table 1).

**Table 1:** The σ^E^ regulon in *S. coelicolor*

**σ^E^ target gene**	**Product**	**Predicted promoter sequence**	**Distance to start codon (bp)**
*sco0662‐0664*	Membrane transport protein; 2‐hydroxyacid dehydrogenase; Hypothetical protein	AGC**AACC**TCGGCTACAACATGGT‐**GGTCT**T	360
*sco0736*	L,D‐transpeptidase	CGC**AACC**AAAGCCGCCGGACGGC‐**GGTCT**A	70
*sco0849‐0848*	Membrane protein; Putative oxidoreductase	AGG**AACG**GATAGGTGTTCTTCGC‐**CATCC**C	306
*sco0877‐0879*	LuxR‐type transcriptional regulator; Hydrolase; AAA domain protein	GCC**AACG**AGGGCCGGACGCCGGC‐**CGTCC**C	18
*sco1023‐1024*	Membrane protein; Hypothetical protein	GGG**AACC**CCGTCCTGGGTCCGCG‐**CGTTG**G	97
*sco1168*	Hypothetical protein	CTG**AACC**TCACGCGCGCGGAGCA‐**CGTCG**T	249
*sco1647*	Hypothetical protein (Pfam: Pup_ligase)	ACC**AACC**CCGACGACTGGGCCCG‐**CATCT**C	362
*sco1738*	Hypothetical protein	TCG**CACG**ACGCCTGCGCGCACAC‐**CGTCT**T	384
*sco1755*	Hypothetical protein	GAC**AACC**AGAAGGCAGGGTTCCG‐**GGTCT**A	38
*sco1875*	HMW PBP	GGG**AACG**ACCGCCGCCCGCCCGTT**CGTCC**T	18
*sco2055*	Membrane protein	CGG**AACC**AATTCTCTCAGACCCG‐**CGTCC**G	171
*sco2168‐2167* (*pspA*)	Phage shock protein A homolog; Hypothetical protein	GGG**AACG**ATCCGGCAACGCCGGT‐**CGTCT**G	262
*sco2255*	Membrane protein	CGG**AACT**CCGCGGGACGGCCCGTA**CGTCC**T	105
*sco2294‐2293*	Putative AraC family transcription regulator; Hypothetical protein (Pfam: EamA)	GGA**CACC**GCGGGATTCCTCTGAT‐**CGTCT**G	23
*sco2334*	Membrane protein	GCC**AACG**TTTCCGTTCGAATTAT‐**CGTCT**T	54
*sco2368*	Hypothetical protein	GGC**AACG**TCTCGCGCGCCTACGG‐**CGTCT**T	317
*sco2419‐2410*	Operon of membrane proteins	TAC**GACC**ACTACTTCAACCTCTT‐**CCTCT**C	224
*sco2611‐2609* (*mreBCD*)	Lateral cell wall biosynthesis	GGG**AACG**GATCCCACCGTTGGCC‐**CGTCT**C	157
*sco2629*	Membrane protein	TTC**AACT**ACAAGTTCCCCGACAC‐**GGTCT**T	171
*sco2807*	Membrane protein	GGC**AACC**CGAGGGGCGATGCCCG‐**CGTCT**A	122
*sco2892*	Membrane protein (Pfam: Lipase_GDSL_2)	CGG**AACG**GAACACAAGTTCCCGG‐**CGTCT**G	113
*sco2897*	HMW PBP, cell wall biosynthesis	GGG**AACG**GAACCCGCGGTGCGAG‐**AGTCT**T	260
*sco2939*	Hypothetical protein	GGC**AACG**AGTGCGTCCCCCCACG‐**CGTCC**T	36
*sco2974* (*pkaA*)	Ser/Thr protein kinase	GGC**AACC**ACGGGACCGGGTCGAG‐**CGTCT**T	108
*sco2975*	Hypothetical protein	CGT**GACC**GATCTCAAGCGGACGG‐**CATTC**G	221
*sco3034* (*whiB*)	Sporulation regulatory protein	CGG**AACG**GGATCGATCGCCGGGG‐**CGTCC**T	238
*sco3044*	LytR‐CpsA‐Psr (LCP) family protein, wall teichoic acid deposition	AGT**GACC**TGAGGGGCCCCGCACG‐**CGTCT**G	335
*sco3098*	Putative secreted protein (Pfam: Transglycosylase, LysM domain)	GTC**AACC**GCCGCGTGGTCCCCGT‐**CGTCT**T	15
*sco3194*	L,D‐transpeptidase, lipoprotein	GGG**AACC**CCACGGGCCGCCGGGCA**CCTCT**A	46
*hrdD* (*sco3202*)	RNA polymerase sigma factor	GGC**AACC**CTCAGGCGGTACGGGC‐**CGTCT**T	375
*sco3342‐3341*	Glycine‐rich secreted protein; Hypothetical protein	GGG**AACG**GTGTGCCGGGCCGAGCG**GCTCT**T	74
*sco3396*	Hypothetical protein (Pfam: Esterase)	CGG**AACC**TCGCCCGACATTTCCT‐**CATCT**G	151
*sco3397* (*mprF*)	Putative MprF lysylphosphatidylglycerol synthase, membrane protein	GTG**AACC**TCTCCCTCCGAGACAC‐**CGTCC**T	95
*sco3419*	Hypothetical protein	CTC**AACG**GCGACACCATGCTGGA‐**CGCCT**T	137
*sco3424*	Putative regulator, similar to AbaA and BldB	GGG**AACG**ACTTCTCGGGCCCCGG‐**CGTCG**T	164
*sco3481*	Hypothetical protein	TGG**AACG**ACTACCTGGTCGCCAC‐**CGTCT**T	207
*sco3548*	Putative anti‐sigma factor	TGC**AACC**AGGAGCGCATTCTCAA‐**GATCT**T	182
*sco3559*	Oxidoreductase	GGG**CACG**GCGCCGGGTTGCGTAG‐**GGTCT**T	4
*sco3712*	Putative hydrolase, similar to polysaccharide deacetylase	GGG**ATCC**CGCGGCGGGTTTCTCC‐**CGTCC**T	5
*sco3728*	Membrane protein	GGG**AACG**GATCGGCGGCCGGCAG‐**CGTCG**T	46
*sco3761*	Hypothetical protein	GGG**AACC**TCGGCATGACCGTGTT‐**CGTCT**C	47
*sco3900‐3899*	Hypothetical protein (Pfam: PadR); Hypothetical protein	CAA**AACC**CCCGCGGCCCGAAGTT‐**CACCT**C	142
*sco3972*	Hypothetical protein (Prim‐Pol domain)	TGG**AACC**CGGCGACGGACCCGGG‐**CGTCC**T	317
*sco4042*	Membrane protein (Pfam: LytR_C)	TCG**AACC**TCGGAACGTCGACTGAT**CATCT**A	60
*sco4069*	Membrane protein	CCG**AACC**CGGCAGGCCCCGGCTC‐**CGTCT**C	259
*sco4120*	Hypothetical protein	AGG**AACT**CCCCCGGCCACCGGGG‐**CGTCT**G	145
*sco4133*	Membrane protein	TGG**AACG**TATCAACGGGGACCGTG**CGTTC**C	84
*sco4134*	Putative lipoprotein	GGG**AACC**CGCGCCCCCACACCCC‐**CGTCT**C	33
*sco4159‐4158*	GlnR transcriptional regulatory protein	GCG**AACC**GGGCACGACCACAAAC‐**CGTCC**C	16
*sco4253*	Hypothetical protein	AGA**AACG**CCGGGCGTCCGCCAGG‐**GGTCT**T	158
*sco4263*	Transcriptional regulator	CAC**CACC**GTTCACCGCAGTCGTT‐**CGTCT**G	38
*sco4289*	Secreted protein	GAC**AACG**TCACGGACGGTTCCCC‐**CGCCT**G	110
*sco4439*	LMW PBP; cell wall biosynthesis	TGG**AACC**AGTAGGTATGTCGTTCT**CGTCT**T	222
*sco4468‐4467*	Hypothetical proteins	GAC**AACC**GCCCCCAACGCCGTGC‐**CGTCT**G	169
*sco4471*	Lipoprotein	CGG**AACC**CGCTCGTTCGTCGCGT‐**CCTCT**C	38
*sco4494*	Hypothetical protein	AGT**AACC**GGGGCGTACCGTTGACC**CGTCT**G	19
*sco4582*	Membrane protein	GGC**AACC**CGACCGGAACCTGTGC‐**CCTCC**C	345
*sco4613*	Membrane protein	CGC**AACC**ACCCGGCGCGGTCGGAA**CGTCC**T	88
*sco4651*	Putative lipoprotein	AGA**AACC**ACAAGATCGTTCGAAC‐**CGTTT**C	105
*sco4847*	LMW PBP; cell wall biosynthesis	CGC**AACC**CGATGACCCCGACGAC‐**CGTCC**C	271
*sco4849*	Membrane protein	GAG**GACG**TCACGGACGCCCTGAG‐**CGTCC**C	20
*sco4904*	Membrane protein (Pfam: VanZ)	CGG**AACC**GCACACGGCGGGGGGCG**CGTCT**A	7
*sco4934*	L,D‐transpeptidase, lipoprotein	GGC**AACC**GCCGCCCGGGGTTTCGT**CGTCT**C	172
*sco4968*	Membrane protein	CGG**AACG**GCGTACCAGCCGCTGAA**GGTCT**A	347
*sco5030*	Membrane protein	CTC**AACC**TCGCGCAGCCCCTCAC‐**CGTCT**T	94
*sco5039*	HMW PBP, cell wall biosynthesis	CAC**AACC**TTGAACCCCGCTCGTA‐**CGTCG**G	335
*sco5049*	Hypothetical protein	GCG**AACT**GTCCGACTTGAATTTCA**CCTTT**C	212
*sco5213*	Membrane protein	GCG**AACC**GGCTCCGGGTCCTCGA‐**CGTCT**T	198
*sco5255*	Signal peptidase protein	GCA**AACA**GGCGGGAAAGCATGAAG**CGTTC**C	132
*sco5310*	Hypothetical protein	GGG**AACG**GGCCGCCACGCGCGCA‐**CGTTC**T	124
*sco5358*	LytR‐CpsA‐Psr (LCP) family protein, wall teichoic acid deposition	TGC**AACC**CTGTCCCGAGTTCCGC‐**CGTCT**G	108
*sco5535‐5336 (accB‐accE)*	Acetyl‐CoA carboxylase complex subunits	TGT**GACC**TCTACAAGCCAGAGGC‐**CCTCT**G	117
*sco5705*	Hypothetical protein	GCG**AACG**CGCTCTCCCCGGCCCG‐**CGTCT**C	304
*sco5742*	Membrane protein	GGG**CACC**TGAAGGGGCGTTCGTT‐**CGTCT**G	49
*sco5856*	Membrane protein	CGG**AACT**AATGGTTTCGGCCGCA‐**CGTCC**C	52
*sco5981*	Hypothetical protein	GCG**AACC**CTCAGCCTCCTCAGAC‐**CCTCT**T	29
*sco6028*	Putative ribonuclease	CGG**AACG**TTCCGTCGGCGGGCTC‐**CGTCG**A	122
*sco6130*	Hypothetical protein (HATPase domain)	CTC**CACC**CCCGTCCCCACGTGAG‐**CGCCT**C	21
*sco6178‐6177*	Putative deacetylase; Hypothetical protein	ATG**AACC**GCGTATATACACGCAG‐**CGTAT**A	50
*sco6179‐6190* (*cwg* operon)	Cell wall glycan synthesis	CGC**AACC**TGGTCCCCGTTTTCGT‐**CGTCT**T	147
*sco6262‐6263*	Putative helicase; Hypothetical protein	CGA**GACC**ACCGGTGCCGGTCTCGA**CGTCT**T	389
*sco6357‐6353*	3 membrane proteins; Response regulator; Sensor kinase	GGG**AACG**TTCCTCACTCCGCCAT‐**CGTCT**A	88
*sco6379*	Membrane protein	TGG**AACG**GTCCTCACCCCGCTGC‐**CGTCT**A	88
*sco6750*	Putative IPP isomerase	GCG**GACG**GCCCGGGGGCGCGCACG**CACCG**G	234
*sco6773*	Putative peptidase (Lysin motif domain)	GGG**AACC**CTTCGCTTGTCCCTGTG**CGTCT**T	224
*sco6832‐6833*	Methylmalonyl‐CoA mutase; isobutyryl‐CoA mutase	GGC**GACC**GTGCTGCGGAGCCCAA‐**CATCT**T	242
*sco6979‐6982*	Solute‐binding lipoprotein; ABC transporter membrane protein; ABC transporter membrane ATP binding protein; Hypothetical protein	CTC**AACC**TCCGCCAGGGGTACGCC**CGTCT**G	322
*sco7233*	Membrane protein	GGC**AACC**CGAAGGATCTCCATCC‐**CCTCC**T	69
*sco7657‐7658*	Membrane protein; Hypothetical protein	GAC**AACC**GGGCATCCGAGCGCTC‐**CCTCT**C	75
*sco7730*	Hypothetical protein	CGA**GACC**GACGCCCCGGCGCGGAC**CATCC**T	245
*scot11*	tRNA‐Met	GGG**AACC**GCGCGGCACGCTGCGG‐**AGTCC**T	107

**Figure 3:**
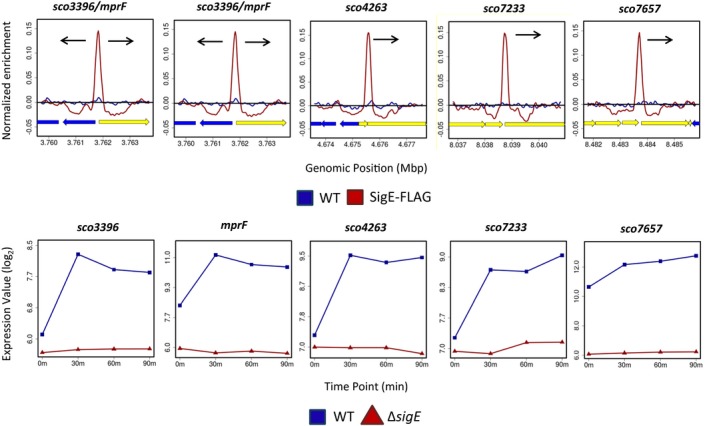
ChIP‐seq (above) and microarray transcriptional profiling data (below) for the Class I σ^E^ target genes *sco3396*, *mprF* (*sco3397*), *sco4263*, *sco7233* and *sco7657*. Class I targets have a single promoter that is completely dependent on σ^E^ for its transcription (see, for example, Fig. 6A). Colour‐coding of the ChIP samples is as follows: *S. coelicolor* M600 (WT, blue), Δ*sigE attB_ΦBT1_*::3 × FLAG‐*sigE* (SigE‐FLAG, red). Plots span approximately 3 kb of DNA sequence. Genes running left to right are shown in yellow, and genes running right to left are shown in blue. The black arrow indicates the gene subject to σ^E^‐dependent transcription. Colour‐coding of the microarray data is as follows: *S. coelicolor* M600 (WT, blue squares), *sigE* null mutant J2130 (∆*sigE,* red triangles). In each panel, the *x*‐axis indicates the time in minutes (0, 30, 60 or 90) after the addition of 10 µg/ml vancomycin, and the *y*‐axis indicates the per gene normalized transcript abundance (log_2_).

**Figure 4:**
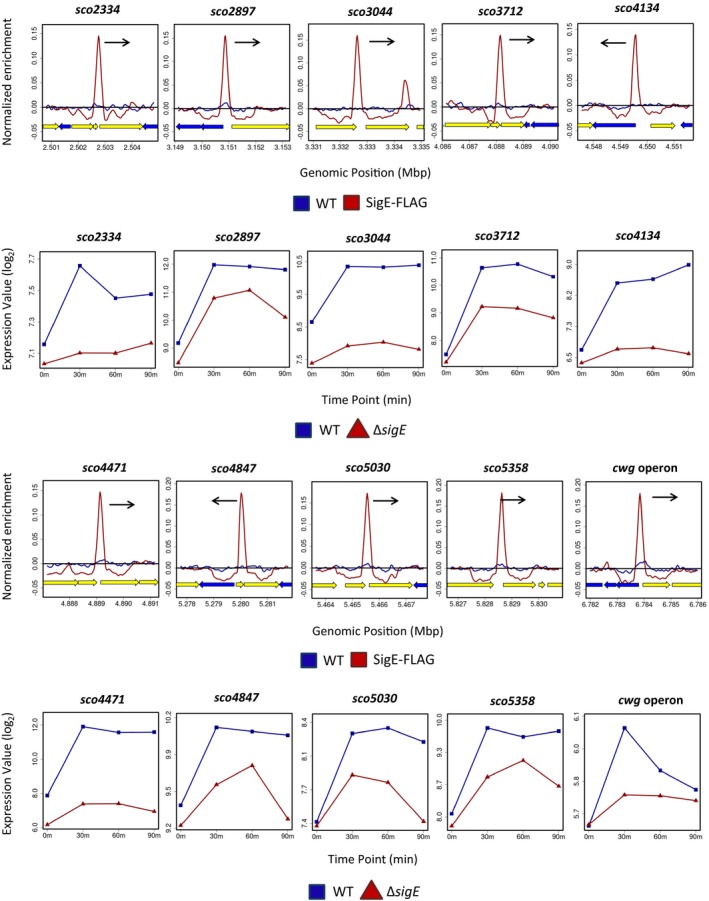
ChIP‐seq (above) and microarray transcriptional profiling data (below) for the Class II σ^E^ target genes *sco2334, sco2897*, *sco3044*, *sco3712*, *sco4134*, *sco4471*, *sco4847*, *sco5030*, *sco5358* and the 12‐gene *cwg* operon (*sco6179‐6190*). Class II targets have a single promoter that is partially dependent on σ^E^ for its transcription (see, for example, Fig. 6B). In the ChIP‐seq panels, the black arrows indicate the genes subject to σ^E^‐dependent transcription. In the microarray transcriptional profiling panels, the *x*‐axis indicates the time in minutes (0, 30, 60 or 90) after the addition of 10 µg/ml vancomycin, and the *y*‐axis indicates the per gene normalized transcript abundance (log_2_). See the legend to Fig. 3 for explanation of the colour‐coding.

**Figure 5:**
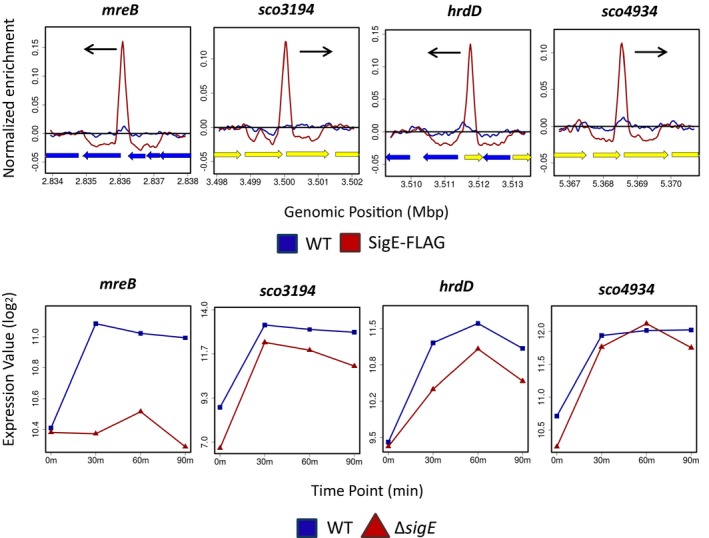
ChIP‐seq (above) and microarray transcriptional profiling data (below) for the Class III σ^E^ target genes *mreB* (*sco2611*), *sco3194*, *hrdD* (*sco3202*) and *sco4934*. Class III targets have multiple promoters, one of which is partially or wholly dependent on σ^E^ (see, for example, Fig. 6C). In the ChIP‐seq panels, the black arrows indicate the genes subject to σ^E^‐dependent transcription. In the microarray transcriptional profiling panels, the *x*‐axis indicates the time in minutes (0, 30, 60 or 90) after the addition of 10 µg/ml vancomycin, and the *y*‐axis indicates the per gene normalized transcript abundance (log_2_). See the legend to Figure 3 for explanation of the colour‐coding.

To determine how σ^E^ influences the expression of its target genes, *S. coelicolor* M600 and the congenic *sigE* mutant were subjected in parallel to time‐resolved, genome‐wide transcriptional profiling following treatment with vancomycin. Note that the transcriptional profiling data for the wild type (but not for the *sigE* mutant) has been published previously (Hesketh *et al.*, 2011). Some σ^E^ ChIP‐seq targets were vancomycin inducible in wild‐type *S. coelicolor* and were completely depended on *sigE* for expression (Figs 3 and S2). However, other σ^E^ ChIP‐seq targets were vancomycin inducible in the wild type and retained vancomycin inducibility to varying degrees in the *sigE* mutant (Figs 4, 5 and S2). This phenomenon was investigated further by analysing the transcription of a selection of genes using S1 nuclease protection assays, covering the full range of σ^E^ ChIP‐seq target genes all the way from those showing complete dependence on *sigE*, such as *sco3396*, *mprF* (*sco3397*), *sco4263* and *sco7657* (Fig. 3), to those showing little or none, such as *sco3194* (Fig. 5).

### Validation and classification of σ^E^ targets by S1 nuclease mapping

**Figure 6:**
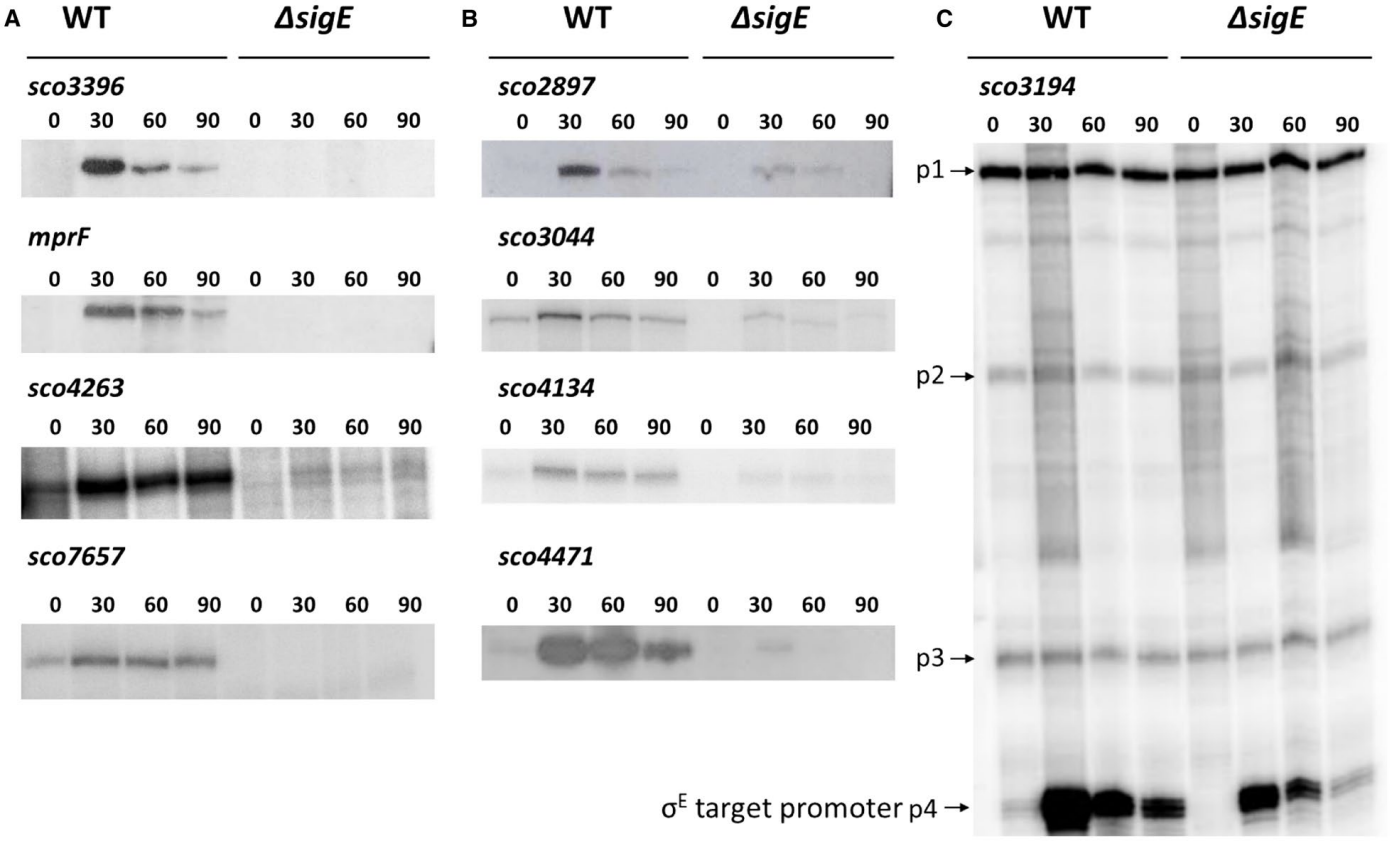
Examples of S1 nuclease protection assays of σ^E^ target genes, divided into three classes. A. Class I genes, having a single promoter that is completely dependent on σ^E^. B. Class II genes, having a single promoter that is partially dependent on σ^E^. (C) Class III genes, having multiple promoters, one of which is partially dependent on σ^E^. RNA was prepared from *S. coelicolor* M600 (WT) and the *sigE* null mutant J2130 (Δ*sigE*) after 0, 30, 60 and 90 minutes treatment with 10 µg/ml vancomycin. In (C), p4 is the σ^E^ target promoter of the *sco3194* gene.

The promoters of 17 σ^E^ target genes [*sco2334*, *mreB* (*sco2611*), *sco2897*, *sco3044*, *sco3194*, *sco3396*, *mprF* (*sco3397*), *sco3712*, *sco4134*, *sco4263*, *sco4471*, *sco4847*, *sco4934*, *sco5030*, *sco5358*, *sco7233* and *sco7657*] were characterised using S1 nuclease protection assays. The results confirmed that the genes identified by ChIP‐seq do indeed dependent upon σ^E^ for their expression (Fig. 6 and data not shown). This was further confirmed by *in vitro* transcription experiments using purified σ^E^ and the promoters of *mreB*, *sco2334*, *sco3194*, *sco3396* and *sco4471* (Fig. S3 and data not shown). Subsequently, we divided the 17 σ^E^ target genes into three classes, based on the number of promoters upstream of each gene and their dependence on σ^E^, as determined by S1 nuclease protection assays (Fig. 6) and the time‐resolved, genome‐wide transcriptional profiling (Figs 3, 4, 5 and S2). Class I genes (*sco3396*, *mprF*, *sco4263*, *sco7233*, *sco7657)* represent targets that have a single promoter that is completely dependent on σ^E^ for its expression (Fig. 6A). In line with the results of the S1 nuclease protection assays, microarray transcriptional profiling showed that the transcription of Class I targets is induced in the presence of vancomycin in the wild type and is entirely dependent upon *sigE* (Fig. 3). Class II genes [*sco2334*, *sco2897*, *sco3044*, *sco3712*, *sco4134*, *sco4471*, *sco4847*, *sco5030*, *sco5358* and the 12‐gene *cwg* operon previously characterised as a σ^E^ target (Hong *et al.*, 2002)] represent targets that have a single promoter that is partially dependent on σ^E^ (Fig. 6B). Once again, in agreement with the S1 nuclease protection assays, microarray transcriptional profiling showed clear induction by vancomycin and partial dependence upon *sigE* (Fig. 4). Finally, Class III genes (*mreB, sco3194*, *hrdD* and *sco4934*) represent targets that have more than one promoter, one of which is partially or wholly dependent on σ^E^ for its expression (Fig. 6C and data not shown). The multiple promoters of *S. coelicolor hrdD* and *mreB* were characterised by S1 nuclease mapping previously (Buttner *et al.*, 1990; Burger *et al.*, 2000). The transcription of these four genes is increased on addition of vancomycin but the dependence on *sigE* is subtle (especially for *sco3194* and *sco4934*). (Fig. 5). Looking across all three classes of σ^E^ target gene, the differences in the number of promoters and the extent of dependence of the σ^E^ target promoter on σ^E^ allows target genes to be expressed with a wide range of induction ratios.

**Figure 7:**
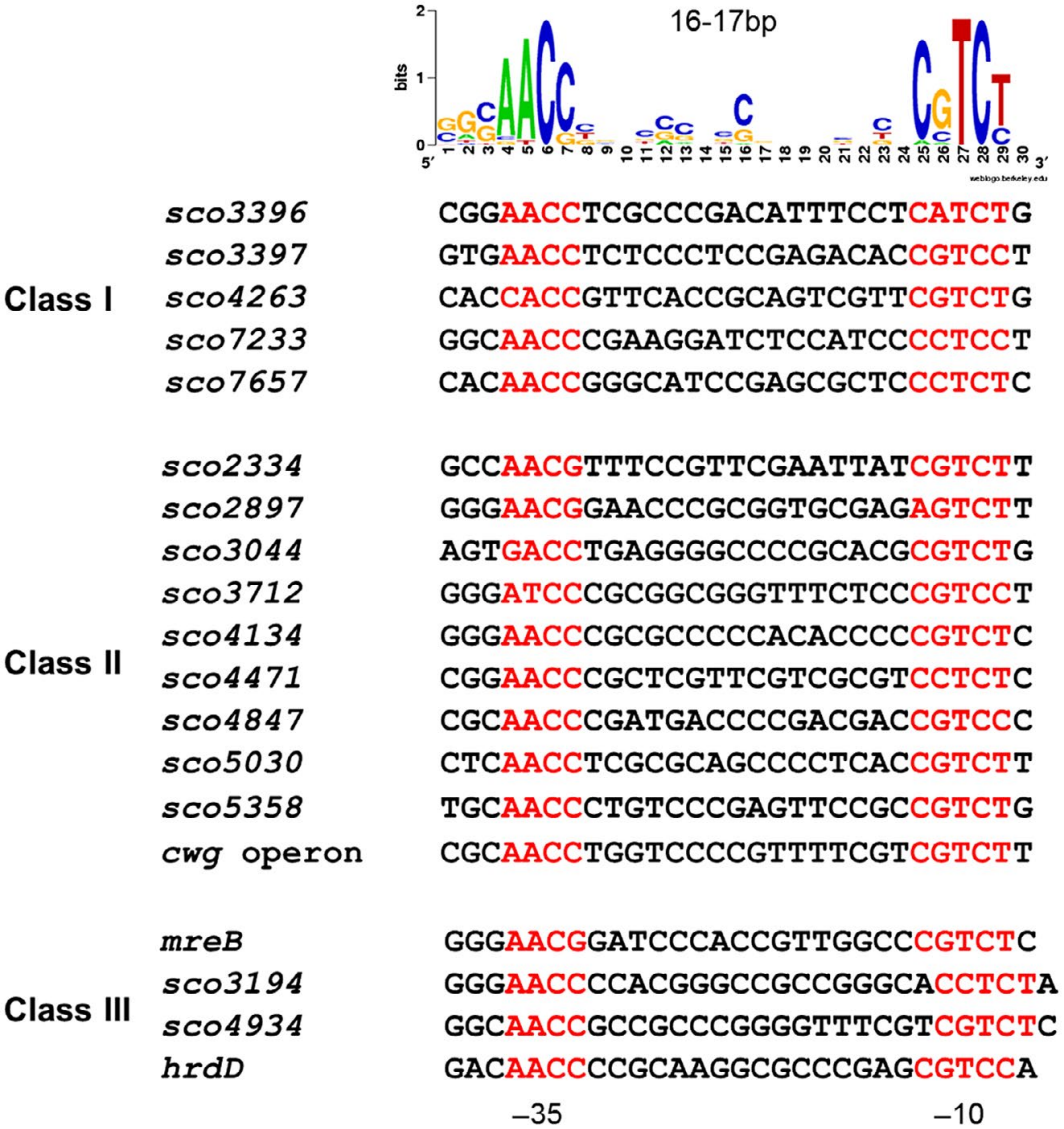
Alignment of the −10 and −35 recognition sequences of the 17 σ^E^ target promoters characterised by S1 mapping, additionally including the previously characterised *hrdD* and *cwg* promoters (Buttner *et al.*, 1990; Hong *et al.*, 2002). The target genes are divided into Class I (one promoter, completely dependent upon σ^E^), Class II (one promoter, partially dependent upon σ^E^) and Class III targets (multiple promoters, one at least partially dependent upon σ^E^). The corresponding σ^E^ consensus sequence, generated using WebLogo (Crooks *et al.*, 2004), is shown above the alignment.

The S1 mapping data were then used to identify the −10 and −35 recognition sequences for the 17 novel targets tested, additionally including the previously characterised *hrdD* and *cwg* promoters (Buttner *et al.*, 1990; Hong *et al.*, 2002) (Fig. 7). Based on these validated promoter sequences, a σ^E^ consensus was generated (Fig. 7) using WebLogo (Crooks *et al.*, 2004). It is noteworthy that no unambiguous distinction exists between the predicted −35 and −10 binding motifs of those promoters that are completely dependent on σ^E^ and those that exhibit partial dependence, although it seems that the latter class are significantly enriched for a G at position 2 of the −10.

The majority of the σ^E^ target promoters tested by S1 mapping are only partially dependent on *sigE*, suggesting that there are additional ECF σ factors that also recognize these promoters. Further, most of these promoters remain vancomycin‐inducible in the *sigE* mutant, implying the additional ECF σ factors involved also respond to cell envelope stress. Overall, these results suggest there is a network of two or more ECF σ factors that cooperate with σ^E^ to maintain cell envelope integrity in *S. coelicolor*, which is perhaps unsurprising given there are 51 ECF σ factors in this species. Overlapping promoter specificity between different ECF σ factors has been described in several bacterial genera. For example, multiple ECF σ factors are involved in the cell envelope stress response in *B. subtilis*, and three of them, σ^M^, σ^W^ and σ^X^, can all contribute to the transcription of a common promoter from the same start site (Mascher *et al.*, 2007; Kingston *et al.*, 2013). The predicted consensus binding motifs for these ECF σ factors are highly similar, with some target promoters belonging to one regulon or the other and other target promoters belonging to more than one regulon (Mascher *et al.*, 2007). It has been shown that single nucleotide changes in the −10 motif can determine whether a given promoter is recognised by σ^X^, σ^W^, or both (Qiu and Helmann, 2001). In addition, it has also been shown that the presence or absence of a homopolymeric T‐tract between the −35 and −10 elements contributes to promoter selectivity between σ^M^, σ^W^, σ^X^ and σ^V^ in *Bacillus* (Gaballa *et al.*, 2018). Finally, there is also a clear analogy with the oxidative stress response in *Streptomyces.* Many promoters in *S. coelicolor* that are recognised by the oxidative stress response σ factor, σ^R^, retain some activity in a *sigR* null mutant and these promoters are frequently still induced by oxidative stress in that background, implying that there is also a network of related ECF σ factors that coordinate the response to oxidative damage in *Streptomyces* (Paget *et al.*, 2001; Kim *et al.*, 2012).

### Genes of the σ^E^ regulon

Over half of the genes under control of σ^E^ encode proteins relating to the cell envelope (Table 1). These proteins include those involved in cell wall peptidoglycan assembly, cell wall teichoic acid deposition, lateral cell wall synthesis and sporulation, as well as membrane modification and maintenance of integrity (Table 1). A further 15 σ^E^ target genes encode proteins involved in signal transduction and gene regulation (including the σ factor, HrdD) emphasizing the pleiotropic role of σ^E^. Indeed, HrdD itself is predicted to regulate the expression of over 80 genes, including a further 31 genes that themselves encode regulatory proteins (Strakova *et al.*, 2014). Hesketh *et al. *(2015) used mass spectrometry to analyse changes in the *S. coelicolor* proteome upon vancomycin‐induced stress. In line with the work presented here, they identified several proteins encoded by σ^E^ target genes that increased in abundance in response to vancomycin treatment, including σ^HrdD^ and the products of *sco1647*, *sco2368* and *sco4494*.

### Cell wall peptidoglycan elongation and assembly

Six σ^E^ target genes encode pencillin‐binding proteins (PBPs). PBPs are involved in the final stage of peptidoglycan synthesis, catalysing its polymerization and cross‐linking outside the membrane (Macheboeuf *et al.*, 2006; Sauvage *et al.*, 2008). PBPs are broadly divided into two classes: the high molecular weight (HMW) PBPs and the low molecular weight (LMW) PBPs. Based on their structure and specific catalytic activity, the HMM PBPs are further subdivided into two classes: A and B (Macheboeuf *et al.*, 2006). Class A enzymes have an N‐terminal glycosyltransferase domain involved in glycan chain elongation and a C‐terminal transpeptidase domain involved in cross‐linking the pentapeptide stems of the glycan units (Macheboeuf *et al.*, 2006; Sauvage *et al.*, 2008). This class of PBP is critical for cell growth in some bacteria such as in *E. coli*, where deletion of the two class A PBPs (PBP1a and PBP1b) is lethal (Denome *et al.*, 1999). Similarly, in *Streptococcus pneumoniae*, deletion of the class A PBPs, PBP1a and PBP2a, appears to be lethal (Hoskins *et al.*, 1999). The σ^E^ target genes *sco2897*, *sco3901* and *sco5039* (*sco3901* is a σ^E^ target in ChIP‐seq but the bioinformatically predicted σ^E^‐binding site is >400 bp upstream, Table S1) encode proteins belonging to this subclass and they are the only three class A HMW PBPs among more than 20 PBPs in *S. coelicolor*. It has been shown that deletion of any of these three PBPs results in decreased vancomycin resistance (Hesketh *et al.*, 2011).

The σ^E^ target *sco1875* encodes a class B HMW PBP and a *sco1875* mutant exhibits increased sensitivity towards both vancomycin and bacitracin (Hesketh *et al.*, 2011). In contrast to class A HMW PBPs, class B HMW PBPs do not include an N‐terminal glycosyltransferase domain, but rather an N‐terminal domain thought to be involved in cell morphogenesis via interaction with partner proteins (Macheboeuf *et al.*, 2006; Sauvage *et al.*, 2008). For example, in *E. coli*, the class B PBP FtsI is recruited by the cell division protein FtsW to the site of cell division (Mercer and Weiss, 2002). In *M. tuberculosis*, a class B HMW PBP (PBPA) is required for cell division and maintenance of cell shape, and phosphorylation of PBPA by the Ser/Thr kinase PknB is suggested to regulate the positioning of PBPA at the cell septum, thereby modulating peptidoglycan synthesis (Dasgupta *et al.*, 2006). Some class B HMW PBPs such as PBP2a from methicillin‐resistant *S. aureus* (MRSA) (Chambers, 1997; Lim and Strynadka, 2002; Katayama *et al.*, 2004) and PBP5fm from *Enterococcus faecium* (Fontana *et al.*, 1994; Sauvage *et al.*, 2002) have a low affinity for penicillin and thus give rise to β‐lactam resistance.

The σ^E^ targets *sco4439* and *sco4847* encode putative D‐ala‐D‐ala carboxypeptidases. These are LMW PBPs involved in the cleavage of the terminal alanine of the pentapeptide stems of the glycan chain and thus modulate peptidoglycan maturation or recycling (Macheboeuf *et al.*, 2006; Sauvage *et al.*, 2008).

Among the six σ^E^ target genes that encode PBPs*, sco2897* and *sco4847* are induced by vancomycin (Fig. 4) and have been confirmed by S1 nuclease protection assays to be transcribed from a single promoter that is partially dependent on σ^E^ (Fig. 6B and data not shown). Microarray transcriptional profiling also shows that *sco1875*, *sco4439* and *sco5039* are induced by vancomycin and that transcription is partially dependent on σ^E^ (Fig. S2). These findings suggest that σ^E^‐directed PBP expression is likely to be an important component of the response to cell envelope damage in *Streptomyces*.

### An alternative pathway to peptidoglycan cross‐linking

The target of β‐lactam antibiotics is the D,D‐transpeptidase activity of HMW PBPs, responsible for the synthesis of 4 → 3 cross‐links between peptide side chains in the peptidoglycan of bacterial cell walls. The σ^E^ targets *sco3194*, *sco4934* (both encoding lipoproteins, the latter secreted through the Tat pathway; Thompson *et al.*, 2010) and *sco0736* encode proteins that contain a L,D‐transpeptidase catalytic domain (Pfam: YkuD). Such proteins cross‐link peptidoglycan by forming 3 → 3 cross‐links between peptide side chains (Hugonnet *et al.*, 2014). This bypasses the typical 4 → 3 transpeptidase activity of PBPs, thus promoting resistance to β‐lactams (Biarrotte‐Sorin *et al.*, 2006). The peptidoglycan of *M. tuberculosis* is rich in 3 → 3 cross links, which are suggested to play a role in the adaptive response of the bacteria during stationary phase (Lavollay *et al.*, 2008). L,D‐transpeptidase activity is also employed by *E. coli* in the attachment of Braun’s lipoprotein (BLP) to the peptidoglycan (Magnet *et al.*, 2007). BLP is involved in cell envelope integrity through the connection of the outer membrane to the peptidoglycan layer (Yem and Wu, 1978; Hayashi and Wu, 1990). Transcription of *sco0736*, *sco3194* and *sco4934* is highly induced by vancomycin and partially dependent on σ^E^ (Figs 5, 6C, S2, and Table S1).

### Cell wall teichoic acid deposition

The σ^E^ targets *sco3044* and *sco5358* encode proteins in the LytR‐CpsA‐Psr (LCP) family and expression of *sco3044* in particular depends heavily on σ^E^ (Figs 4 and 6B). LCP proteins are involved in the attachment of wall teichoic acid (WTA) and capsular polysaccharides to the peptidoglycan of the bacterial cell wall (Kawai *et al.*, 2011). WTA can constitute up to 60% of the Gram‐positive cell wall and has roles in the regulation of cell division, cell shape determination, antibiotic resistance and pathogenesis (Brown *et al.*, 2013). In *B. subtilis*, there are three LCP homologs and deletion of all three genes results in a failure to deposit WTA at the cell envelope (Kawai *et al.*, 2011). Similarly, deletion of all three LCP genes in *Staphylococcus aureus* leads to release of WTA into the extracellular medium (Chan *et al.*, 2013) and abnormalities in septum placement and cell separation (Over *et al.*, 2011). Transcription of the LCP gene *msrR* in *S. aureus* is induced by cell wall disrupting agents such as β‐lactams, glycopeptides and lysostaphin, and deletion of *msrR* results in increased sensitivity to methicillin and teicoplanin (Rossi *et al.*, 2003). These observations implicate the σ^E^ response in the maintenance of cell wall components other than peptidoglycan in *Steptomyces*.

### The cytoskeleton, cell wall synthesis and sporulation

Unexpectedly,* mreB* was found to be a σ^E^ target (Fig. 5). MreB is an actin homolog that acts in rod‐shaped bacteria like *E. coli, B. subtilis* and *Caulobacter crescentus* as a cytoskeletal element to direct peptidoglycan biosynthesis in the lateral wall (Errington, 2015). However, in contrast to rod‐shaped bacteria, *Streptomyces* hyphae do not grow by inserting new cell wall material in the lateral wall, but rather by tip extension and by initiating new branches *de novo*. This polar mode of growth does not require MreB and is instead directed by a polarisome complex involving DivIVA, Scy and FilP (Bush *et al.*, 2015). Rather, MreB appears to direct spore wall thickening, localizing under the membrane at spore septa at cell division before spreading around the immature spore (Mazza *et al.*, 2006; Kleinschnitz *et al.*, 2011). However, *mreB* is abundantly transcribed during vegetative growth (Fig. 5) (Burger *et al.*, 2000), suggesting that MreB might have an additional role unconnected to sporulation. *S. coelicolor mreB* mutants sporulate poorly and overproduce actinorhodin (Mazza *et al.*, 2006), and the *sigE* null mutant exhibits similar characteristics (Paget *et al.*, 1999a).

This study also identified the *whiB* gene, encoding the key developmental transcription factor WhiB, as a σ^E^ target. WhiB is essential for the initiation of sporulation septation in *Streptomyces* (Bush *et al.*, 2015; 2016; Bush, 2018), and in *M. tuberculosis* the expression of the WhiB ortholog WhiB2 is induced by cell wall‐inhibiting agents (isoniazid, ethambutol and cycloserine) (Geiman *et al.*, 2006). *S. coelicolor whiB* has two promoters, and the upstream promoter was previously shown to be recognised by σ^E^ in a run‐off assay (Soliveri *et al.*, 1992; Kang *et al.*, 1997). The transcription of *whiB* is highly induced by vancomycin in a σ^E^‐dependent manner (Table 1). Unexpectedly, these observations suggest that *whiB* might play a significant role in the σ^E^‐mediated cell envelope stress response.

### Membrane modification

The σ^E^ target *sco3397* encodes a homolog of *B. subtilis* MprF (multiple peptide resistance factor) (24% identity, 50% similarity) (Table 1). Transcription of *sco3397* is highly induced by vancomycin and is completely dependent on σ^E^ (Figs 3 and 6A). MprF proteins are lysylphosphatidylglycerol synthases that catalyse the transfer of L‐lysine from lysyl‐tRNA to the negatively charged lipid phosphatidylglycerol, thus neutralizing the membrane surface charge. This enhances resistance to cationic antimicrobial peptides (CAMPs) and antibiotics through repulsion (Ernst *et al.*, 2009). MprF has also been shown to affect resistance to vancomycin and daptomycin in *S. aureus* (Ruzin *et al.*, 2003; Nishi *et al.*, 2004; Friedman *et al.*, 2006). In *S. coelicolor*, a *sco3397* mutant shows markedly increased sensitivity towards both vancomycin and bacitracin (Hesketh *et al.*, 2011), in line with a role in the cell envelope stress response. In addition to Sco3397, there is a second homolog of *B. subtilis* MprF in *S. coelicolor* (Sco6384), but the *sco6384* gene is not a σ^E^ target.

### Maintenance of membrane integrity

The σ^E^ target *sco2168* encodes a PspA (phage shock protein A) homolog (Vrancken *et al.*, 2008). PspA is the major effector of the phage shock protein (Psp) system present in many bacteria. The Psp system plays a role in the adaptive response to multiple extracytoplasmic stresses, blocking stress‐induced membrane damage and the resulting dissipation of the proton‐motive force (Joly *et al.*, 2010). In *Streptomyces lividans*, the *pspA* gene is strongly induced under stress conditions that attack membrane activity and is essential for growth and survival under most of these conditions (Vrancken *et al.*, 2008). Both PspA and its paralog LiaH are induced as part of the cell envelope stress response in *B. subtilis* (Jordan *et al.*, 2008). While PspA is under control of σ^W^, LiaH is the primary target of the LiaRS two‐component system and is strongly induced by antibiotics targeting the membrane‐anchored steps of cell wall biosynthesis (Wiegert *et al.*, 2001; Wolf *et al.*, 2010).

### The σ^E^ target *sco4471* encodes a novel lipoprotein that contributes to lysozyme resistance

The σ^E^ target gene *sco4471* encodes a lipoprotein (Thompson *et al.*, 2010) and is heavily but not completely dependent on *sigE* for its transcription (Figs 4 and 6B). Furthermore, *sco4471* expression increases dramatically in response to induction, being more than 20‐fold higher in wild‐type *S. coelicolor* than the *sigE* mutant after treatment with vancomycin (Fig. 4 and Table S1). Deletion of *sco4471* resulted in a fourfold increase in sensitivity to lysozyme compared to wild type (Fig. S4), suggesting that loss of *sco4471* expression contributes to the ~50‐fold increase in lysozyme sensitivity seen in the *sigE* mutant relative to the wild type (Paget *et al.*, 1999a). As shown in Fig. S4, the *sco4471* mutant also displays minor abnormalities in spore size and shape.

### Conservation of σ^E^ target promoters across the *Streptomyces* genus

Following our identification of the genes under σ^E^ control in *S. coelicolor*, we searched bioinformatically to determine if the promoters of these σ^E^ target genes were conserved across the panel of 19 *Streptomyces* species listed in Table S2. Given the high conservation of the σ_2_ and σ_4_ domains (that bind the −10 and −35 promoter elements respectively) in the σ^E^ orthologs across the 19 species (Fig. S5), we anticipated that these 19 σ^E^ orthologs would recognize highly similar or identical promoter motifs to *S. coelicolor* σ^E^. Accordingly, two promoter position weight matrices (PWMs) with a 16 bp or 17 bp spacer between the −35 region and −10 region were generated from 19 validated σ^E^ *S. coelicolor* target promoter sequences. These two PWMs were then used to predict all possible σ^E^‐binding sites that lie within 10–200 bp of the start codon of the downstream gene across the 19 genomes. In *S. coelicolor*, this prediction detected each of the 19 *in vitro* validated σ^E^ targets and over 70% of the targets identified in our ChIP‐seq experiments (Table 1), suggesting suitable parameters for accurate prediction.

**Figure 8:**
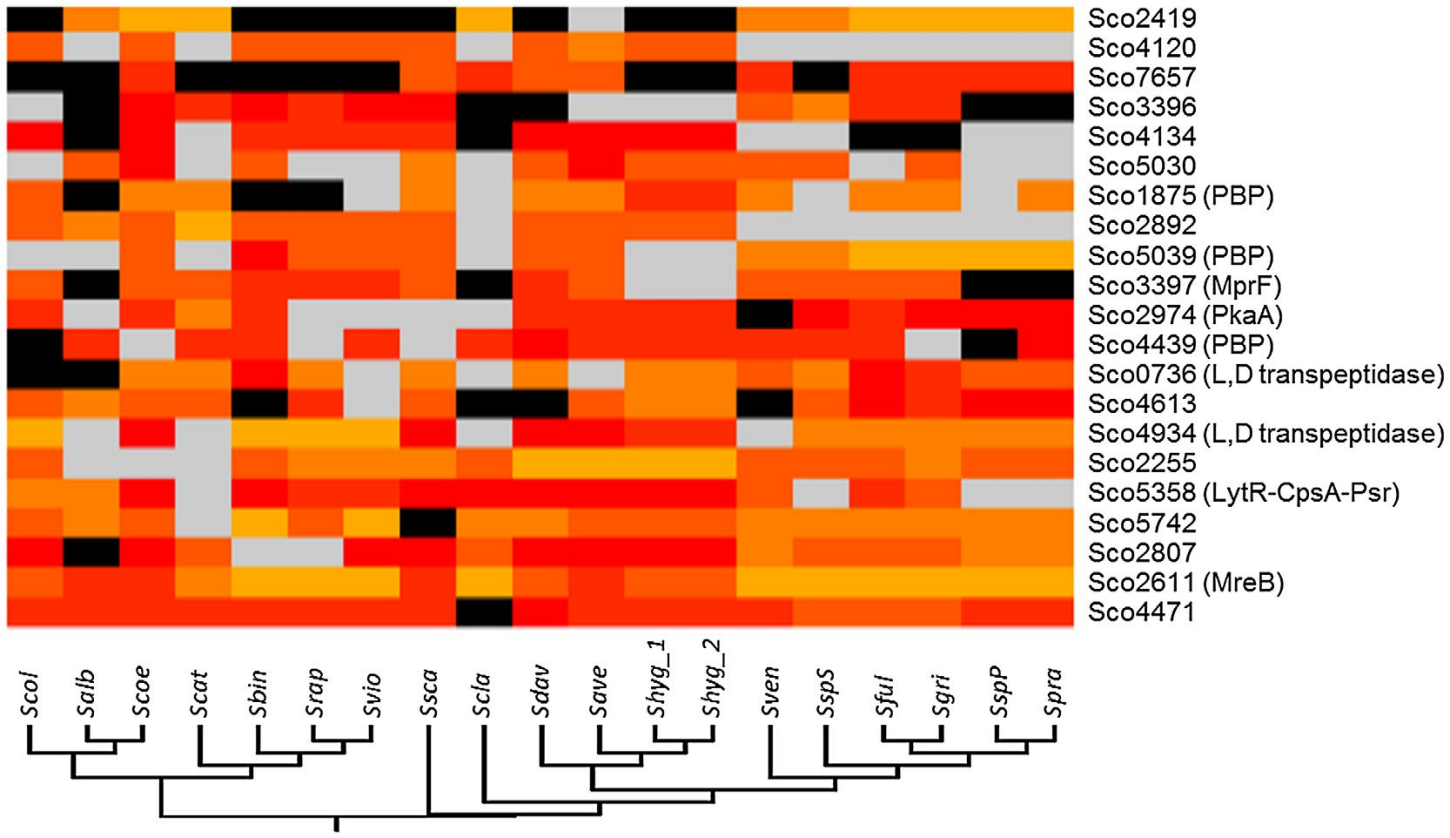
Bioinformatic analysis of the conservation of *S. coelicolor* σ^E^ target promoters across 19 *Streptomyces* genomes, showing the 21 *S. coelicolor* σ^E^ target promoters that are predicted to be conserved in at least 9 *Streptomyces* genomes. Black indicates no ortholog of the target gene is found in the designated genome. Grey indicates the ortholog of the target is found, but the σ^E^‐binding consensus is not present between within 200 bp upstream of the open reading frame. Yellow, orange and red indicate that an ortholog of the target is found and that there is a σ^E^‐binding consensus within 200 bp upstream of the open reading frame. The σ^E^‐binding consensus of each target was predicted by the Virtual Footprint version 3.0 tool incorporated into the PRODORIC server (http://www.prodoric.de/vfp/vfp_regulon.php) (Münch *et al.*, 2005; Grote *et al.*, 2009) and a PRODORIC score was given to reflect the quality of the prediction. The yellow to red linear gradient indicates the Prodoric score of the σ^E^‐binding site from the minimum value to the maximum value. The abbreviations used for each species are the same as those listed in Table S2. The phylogenetic relationship between these *Streptomyces* strains is shown by the phylogenetic tree of their 16s rDNA at the bottom.

This analysis predicts that 21 of the 91 σ^E^ target promoters identified in *S. coelicolor* are conserved across at least 9 of the 19 *Streptomyces* genomes (Fig. 8). These 21 genes (equivalent to *sco0736*, *sco1875*, *sco2255*, *sco2419*, *mreB*, *sco2807*, *sco2892*, *pkaA*, *sco3396*, *mprF*, *sco4120*, *sco4134*, *sco4439*, *sco4471*, *sco4613*, *sco4934*, *sco5030*, *sco5039*, *sco5358*, *sco5742*, *sco7657*) include 9 targets (*mreB*, *sco3396*, *mprF*, *sco4134*, *sco4471*, *sco4934*, *sco5030*, *sco5358* and *sco7657*) validated in our S1 mapping and *in vitro* transcription experiments. *mreB* is present in all 19 predicted σ^E^ regulons, and the gene encoding the lipoprotein that contributes to lysozyme resistance, *sco4471*, is present in 18/19 predicted σ^E^ regulons. Also among the products of these 21 genes are the PBPs Sco1875, Sco4439 and Sco5039, the L,D‐transpeptidases Sco0736 and Sco4934, the putative MprF protein Sco3397, and the LytR‐CpsA‐Psr family protein Sco5358. *sco2255*, *sco2892, sco3396, sco4134* and *sco7657* encode cell envelope‐associated enzymes, whereas *sco2419*, *sco2807, sco4613, sco5030* and *sco5742* encode cell envelope proteins of completely undefined function (Table 1). Finally, PkaA (Sco2974) is a Ser/Thr protein kinase and Sco4120 is predicted to be a regulatory protein (Table 1).

### Conclusions

**Figure 9:**
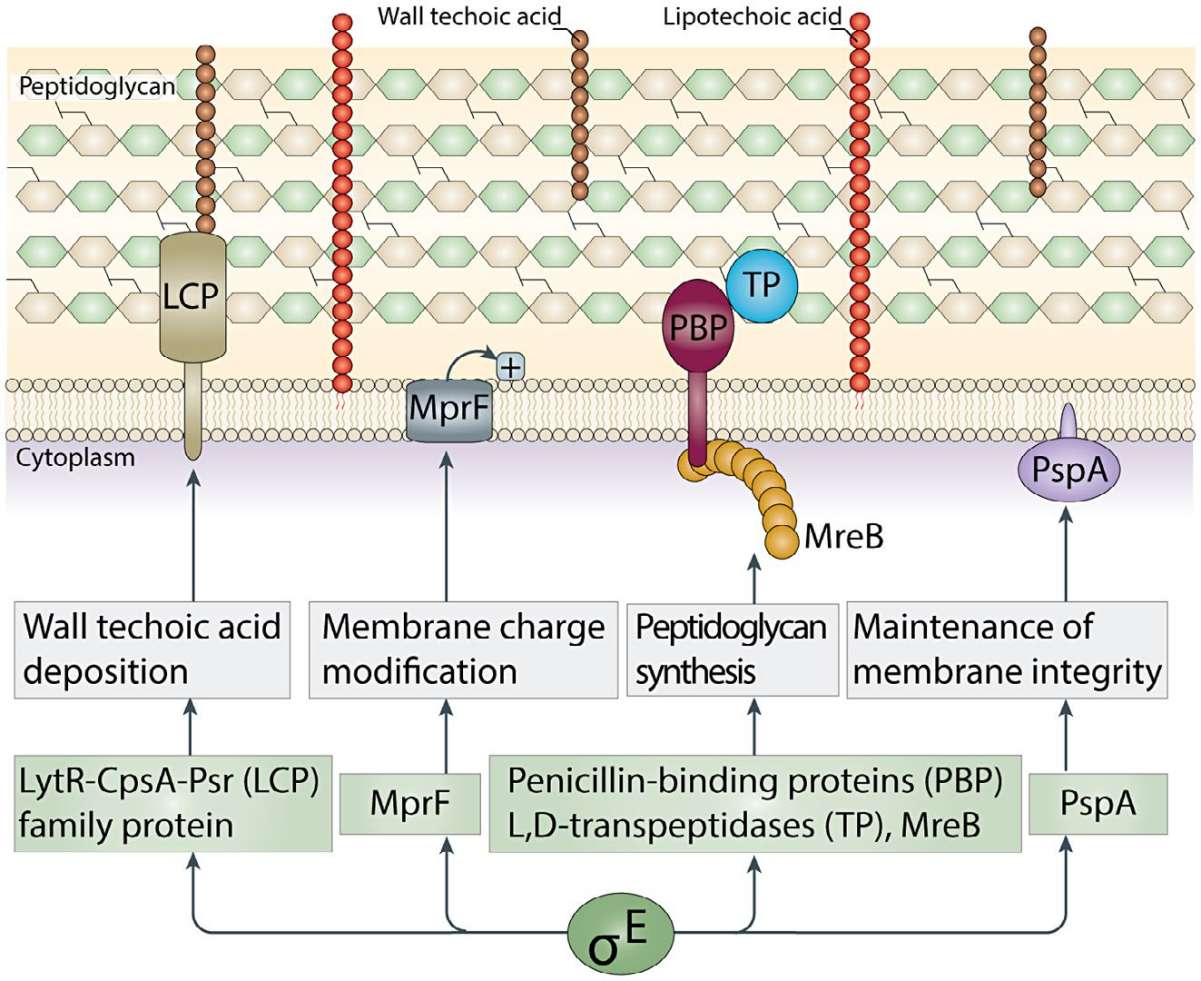
Mechanisms underlying the σ^E^‐dependent cell envelope stress response. Key proteins under σ^E^ control include the actin homolog MreB, multiple PBPs and L,D‐transpeptidases, a LytR‐CpsA‐Psr family protein predicted to be involved in cell wall teichoic acid deposition, PspA, involved in the maintenance of membrane integrity, and a predicted MprF protein that adds lysyl groups to phosphatidylglycerol to neutralize membrane surface charge, potentially contributing to resistance to cationic antimicrobial peptides and antibiotics.

This study reveals the complex regulatory network activated by σ^E^ in response to cell envelope‐induced stress (Fig. 9). In particular, it shows that key proteins under σ^E^ control include the actin homolog MreB, multiple PBP and L,D‐transpeptidases, a LytR‐CpsA‐Psr‐family protein involved in cell wall teichoic acid deposition, PspA, involved in the maintenance of membrane integrity, and a putative MprF protein, predicted to add lysyl groups to phosphatidylglycerol to neutralize membrane surface charge, potentially contributing to resistance to cationic antimicrobial peptides and antibiotics (Fig. 9).

## Experimental proceedures

### Bacterial strains, plasmids and oligonucleotides

Bacterial strains, plasmids and primers in this study are listed in Table S3.

### Construction of a 3 × FLAG‐σ^E^‐complemented *S. coelicolor* strain

In order to engineer an *S. coelicolor* strain expressing a form of σ^E^ with an N‐terminal, triple‐FLAG tag (DYKDHDGDYKDHDIDYKDDDDK), a pMS82‐derived construct was created via a two‐step fusion PCR approach. In the first step, the cosmid STE94 was used as a template for two separate PCR reactions. The first reaction amplified the promoter region of the *sigE* gene using the primer pair P1_3NFLAGSigE_ and P2_3NFLAGSigE_. The second reaction amplified the coding region of the *sigE* gene using the primer pair P3_3NFLAGSigE_ and P4_3NFLAGSigE_. Together the P2_3NFLAGSigE_ andP3_3NFLAGSigE_ primers contain the sequence encoding the triple‐FLAG tag via a 24bp overlapping section. In the second step, the primers P1_3NFLAGSigE_ and P4_3NFLAGSigE_ were used to amplify the entire *sigE* gene and its promoter, fusing the two products from step 1 together and incorporating the 3 × FLAG tag sequence between them. The P1_3NFLAGSigE_ and P4_3NFLAGSigE_ primers additionally contain the HindIII and KpnI sites, respectively, to enable cloning into HindIII, KpnI‐cut pMS82. The resulting vector was then introduced into the Δ*sigE* mutant J2130 (Paget *et al.*, 1999a) by conjugation using the *dam dcm hsdS E. coli* strain ET12567 containing pUZ8002.

### Lysozyme sensitivity tests

Lysozyme sensitivity tests for the wild‐type strain M600, the *sigE* mutant J2130 and the 3 × FLAG‐σ^E^‐complemented *sigE* mutant strain were performed as described previously (Paget *et al.*, 1999a). Briefly, 2×10^6^ spores of *S. coelicolor* were spread onto a Difco Nutient Agar (DNA) plate to make a confluent lawn. 5 µl of 1 mg/ml lysozyme was then diluted in a twofold series and spotted onto the freshly spread spore lawns before incubation at 30°C for 2 days. Lysozyme sensitivity tests were carried out on the wild‐type strain M600 and the *∆sco4471* mutant in the same way but using lysozyme concentrations ranging from 3.75 to 0.0075 mg/ml, generated as a twofold dilution series.

### Western blot analysis

The 3 × FLAG‐*sigE* complemented *sigE* mutant was incubated in 5 ml TES buffer (250 mM N‐[tris(hydroxymethyl)methyl]‐2‐aminoethanesulfonic acid, pH7.2) for 10 minutes at 50°C and germination carried out in 5 ml 2 × PG (0.5 ml of 10% yeast extract, 0.25 ml of 20% casamino acids, 0.05 ml of 1M CaCl_2_ and 4.2 ml of H_2_O) medium for 2–3 hours. Following this, germinated spores were span down at 4500 × g for 10 minutes and inoculated into 50 ml NMMP medium in 250 ml canonical flasks with springs to achieve a final OD_450 _of 0.010, then grown at 30°C, shaking at 250 rpm. At OD_450_ ~ 0.6, vancomycin was added to a final concentration of 10 µg/ml and samples were collected at 15 minutes intervals for 1 hour.

For Western blotting, for each time point, 5 ml culture was taken and spun down at 3000 rpm for 1 minutes. Cells were washed in 5 ml ice‐cold sonication buffer [20 mM Tris pH 8.0, 5 mM EDTA, 1 × EDTA‐free protease inhibitors (Roche)] and finally resuspended in 1 ml before sonication (5 × 5 seconds on, 15 seconds off) at 4.5 micron amplitude. Lysates were then centrifuged at 16,000 × g for 15 minutes at 4°C to remove cell debris. Total protein concentration was determined using the Bradford assay (Biorad). Equal amounts of total protein from each sample were loaded on a 12.5% polyacrylamide SDS‐PAGE gel. After electrophoresis, transfer was carried out to a Hybond‐C Extra nylon membrane (Amersham Pharmacia Biotech) using the Invitrogen XCell II Blot system. For detection of 3 × FLAG‐σ^E^, anti‐σ^E^ polyclonal antibody raised in rabbit was diluted in a ratio of 1:300. 3 × FLAG‐σ^E^ was visualised via an anti‐rabbit IgG alkaline phosphatase secondary antibody (sigma A8025), diluted 3:5000 and detected directly on the membrane using the SigmaFast system (Sigma) that uses BCIP/NBT (5‐Bromo‐4‐chloro‐3‐indolyl phosphate/Nitro blue tetrazolium) as a substrate.

### RNA isolation and DNA microarray analysis

RNA isolation from *S. coelicolor* was performed as described previously (Hong *et al.*, 2002). Total RNA was isolated from mycelium harvested from 5 ml liquid cultures using an RNeasy Midi Kit (Qiagen) according to the manufacturer’s instruction with some modifications. The cell pellet was resuspended in TE buffer containing lysozyme (10 mM Tris, pH 8, 1mM EDTA, 15 mg/ml lysozyme) and incubated at room temperature for 60 minutes. RLT buffer (Qiagen) was added (4 ml) and samples were sonicated 3 cycles ON‐OFF on ice at 18 micron amplitude and for 20 seconds. Samples were then extracted twice with Phenol:Chloroform:Isoamyl Alcohol 25:24:1 saturated with 10 mM Tris, pH 8.0, 1 mM EDTA (2 ml) and once with chloroform (4 ml). Extracts were mixed with 100% ethanol and applied to RNeasy Midi columns. Purified RNA was eluted with 300 µl RNase‐free water. Affymetrix Gene Chip hybridization and data collection were essentially as described before (Hesketh *et al.*, 2009; Bibb *et al.*, 2012). The CEL files received from the scanner were read into the R package for statistical computing (R Core Team, 2012) using the *ReadAffy* function of the *affy* package (Gautier *et al.*, 2004). The *rma* function of the *affy* package was used to compute an *ExpressionSet* object from the CEL files. This *ExpressionSet* object contains the expression values (log_2_) for each gene in each CEL file. The function *lmFit* of the *limma* package (Smyth, 2005) along with a suitable design matrix, was used to combine replicate arrays into single coefficients of expressions for each gene at each time point or strain into an *MArrayLM* object. Expression values were retrieved from the *MArrayLM* object and subjected to a per gene normalization to the median before being used to generate the graphs shown in this paper.

### Chromatin immunoprecipitation sequencing

Spores of the* S. coelicolor* wild‐type strain M600 and the congenic 3 × FLAG‐σ^E^‐complemented *sigE* mutant spores were germinated and grown as described for the Western blot analysis. For the Chromatin immunoprecipitation (ChIP), the cell envelope stress response was induced by treatment with vancomycin to a final concentration of 10 µg/ml and for 30 minutes. Following this, formaldehyde was added to cultures at a final concentration of 1% (v/v) and incubation was continued at 30°C with shaking for a further 30 minutes. Glycine was then added to a final concentration of 125 mM to stop the cross‐linking. Cells were then harvested, lysed, sonicated and the immunoprecipitation conducted via M2 (Sigma Aldrich A2220) gel suspension. Subsequent steps were conducted as described by Bush *et al. *(2013). Notably, for each tested strain, while immunoprecipitated DNA was used as a ChIP (input) sample, the non‐immunoprecipitated total DNA was used as a reference sample. Sequence analysis was conducted as described by Bush *et al. *(2013).

### Data availablilty

The anti‐FLAG‐σ^E^ ChIP‐seq data and microarray transcriptional profiling data have been deposited at the MIAME‐compliant ArrayExpress database (https://www.ebi.ac.uk/arrayexpress/) under accession numbers E‐MTAB‐7827 (ChIP‐seq data) and E‐MTAB‐7814 (microarray transcriptional profiling data).

### S1 nuclease mapping

To generate the probes, a reverse primer within 80 bp downstream of the startcodon of each gene was first labelled with [ɣ‐32P] ATP. Amplification was then conducted from a template using the labelled reverse primer and a forward primer 400 bp upstream of the start codon. For all assays, 30 μg of RNA and 25 pmol of labelled probe were dissolved in 20 μl of sodium TCA buffer and hybridized at 45°C overnight after denaturation at 65°C for 15 minutes. Primer sequences used in S1 nuclease mapping are listed in Table S3. Sequencing ladders were generated using Sequenase™ Version 2.0 DNA Sequencing Kit (USB Europe GMBH).

### Purification of σ^E^ and *in vitro* transcription assays

σ^E^ was overexpressed and purified to homogeneity as described previously (Paget *et al.*, 1999a). Run‐off transcription assays were performed using [α‐^32^P]‐CTP (Perkin Elmer) at 3000 Ci mmol^−1^ as described previously (Buttner *et al.*, 1987). Reaction mixtures contained 1.25 pmol of *E. coli* core RNA polymerase (Epicentre Technologies) and 6 pmol of σ^E^. Transcripts were analysed on a 6% (w/v) polyacrylamide‐7 M urea gel using a heat‐denatured, ^32^P‐labelled *Hpa*II digest of pBR322 as size standards. Cold RNase‐free PCR probes generated as for the S1 mapping experiments were used as templates.

### Construction of a *sco4471* mutant

A* sco4471* mutant in which the coding region was replaced with an apramycin resistance (*apr*) cassette was generated using ‘Redirect’ PCR targeting (Gust *et al.*, 2003). Cosmid D65 was introduced into *E. coli* BW25113 containing pIJ790 and the relevant gene was replaced with the *apr‐oriT* cassette amplified from pIJ773 using the primer pair SCO4471KOFW and SCO4471KORV (Table S3). The resulting disrupted cosmid was confirmed by restriction digestion and by PCR analysis using appropriate flanking primers (Table S3) and introduced into *S. coelicolor* by conjugation via the methylation‐deficient *E. coli* strain ET12567 (*dam dcm hsdS*) carrying the driver plasmid pUZ8002. Null mutant derivatives, generated by double crossing over, were identified by their apramycin‐resistant, kanamycin‐sensitive phenotypes, and their chromosomal structures were confirmed by PCR analysis using appropriate flanking primers (Table S3) and by Southern hybridization.

### Scanning electron microscopy of the *S. coelicolor sco4471* mutant

For scanning electron microscopy, 5‐day‐old colonies were mounted on the surface of an aluminium stub with optimal cutting temperature compound (BDH Laboratory Supplies, Poole, England). The stub was then immediately plunged into liquid nitrogen slush at approximately −210°C to cryopreserve the material and transferred to the cryostage of an ALTO 2500 cryotransfer system (Gatan, Oxford, England) attached to a Zeiss Supra 55 VP field emission gun scanning electron microscope (Zeiss SMT, Germany). The surface frost was sublimated at −95°C for 3 minutes before the sample was sputter‐coated with platinum for 2 minutes at 10 mA at below −110°C. After sputter‐coating, the sample was moved onto the cryo stage in the main chamber of the microscope, held at approximately −130°C. The sample was imaged at 3kV and digital TIFF files were stored.

### Prediction of the promoter motif associated with each ChIP‐seq target

Initially, at least 200 bp sequences surrounding the highest enriched ‘25 bp’ genomic region of all the ChIP‐seq targets were extracted. Then, over‐represented 2‐block motifs mimicking a typical promoter with conserved ‘‐35’ and ‘‐10’ regions were identified in the forward strand of these sequences by the BioProspector program using the parameters: ‘W = 4’, ‘w = 5’, ‘G = 17’, ‘g = 16’ and ‘G‐g = 1 bp’ (‘W’ and ‘w’ stand for the length of the upstream and downstream motifs respectively; ‘G’ and ‘g’ stand for the maximum and minimum distances between the two blocks respectively) (Liu *et al.*, 2001). The 2‐block motifs were obtained from iterative searches using all combinations of these parameters. After 40 reinitializations, the highest scoring motifs were then selected to represent the σ^E^‐binding sites since they highly resemble the previous reported σ^E^ promoter motif.

### Bioinformatic analysis of the conservation of *S. coelicolor* σ^E^ target promoters across 19 *Streptomyces* genomes

Two promoter PWMs, PWM_19_16 and PWM_19_17, were built from the 19 validated *S. coelicolor* σ^E^ promoter sequences shown in Figure 7 by restricting the spacer between the −35 region and −10 region to be 16 bp and 17 bp respectively. In the case of PWM_19_16, one base was removed from the non‐conserved region of the *sco3194* and *sco4934* promoters respectively, whereas, in the case of PWM_19_17, one base was added into the non‐conserved region of each promoter with 16 bp between the −35 region and −10 region. Then, these promoter PWMs were used to search for putative σ^E^‐binding sites from 19 *Streptomyces spp.* chromosome sequences using the Virtual Footprint version 3.0 tool incorporated into the PRODORIC server (http://www.prodoric.de/vfp/vfp_regulon.php) (Münch *et al.*, 2005; Grote *et al.*, 2009) with the parameters: ‘Non‐Occurrence Penalty = None’, ‘Sensitivity = 1’, ‘Core Sensitivity/Size = 1/6’. Searches were restricted to sequences between 10 and 200 bp upstream from the start codon of the closest predicted coding sequence. Orthologues of these targets were searched for in each *Streptomyces* genome using BlastP. *S. coelicolor* σ^E^ target promoters predicted to be conserved in at least 9 of the 19 genomes analysed are shown in Figure 8.
