# Supplementary material for: Defining the regulon of genes controlled by σE, a key regulator of the cell envelope stress response in Streptomyces coelicolor
Source: Mol Microbiol. 2019 Apr 29;112(2):461–81. doi: 10.1111/mmi.14250 (PMC6767563; doi:10.1111/mmi.14250)
Supplement: Supplementary file 1 [file MMI-112-461-s001.pdf]

SUPPORTING INFORMATION

Defining the regulon of genes controlled by σ^E, a key regulator of the cell envelope stress response in *Streptomyces coelicolor*

Ngat T. Tran, Xiaoluo Huang, Hee-Jeon Hong, Matthew J. Bush, Govind Chandra, Daniela Pinto, Maureen J. Bibb, Matthew I. Hutchings, Thorsten Mascher, and Mark J. Buttner*

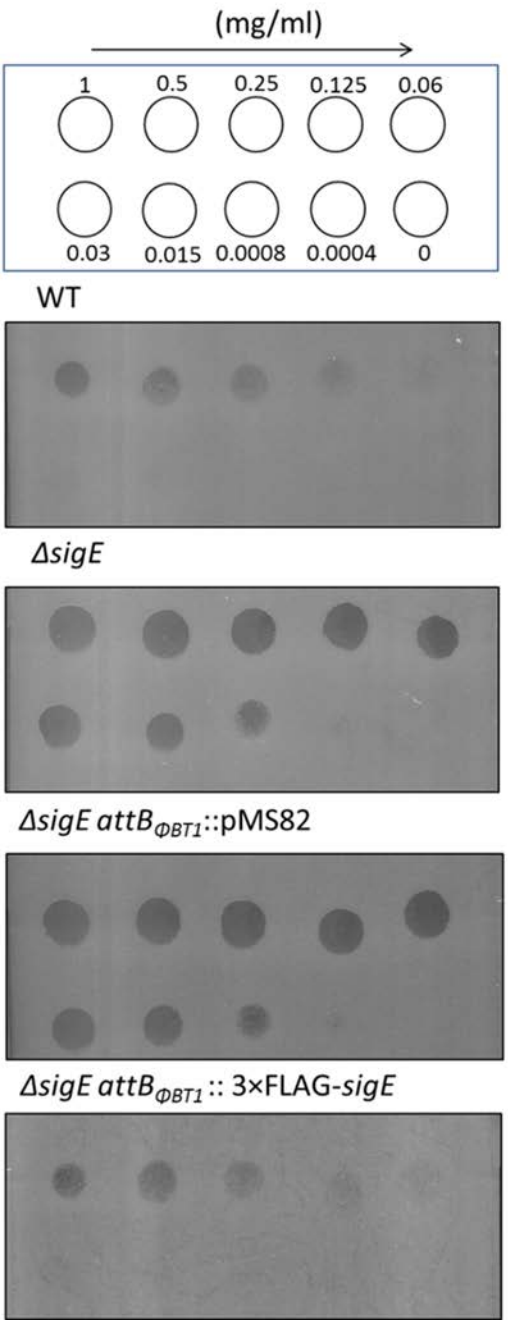

Fig. S1. Lysozyme sensitivity of wild-type *S. coelicolor* M600 (WT), the *sigE* null mutant J2130 (*ΔsigE*), J2130 harbouring the empty pMS82 vector (*ΔsigE attB$_{ΦBT1}$*::pMS82), and J2130 expressing the N-terminal, triple FLAG-tagged $σ^E$ (Δ*sigE attB$_{ΦBT1}$*:: 3×FLAG-*sigE).* 5 µl lysozyme in a 2-fold dilution series was spotted onto DNA agar immediately after plating $2×10^6$ spores of each strain to create a confluent lawn. Images were taken after 2 days of growth at 30ºC.

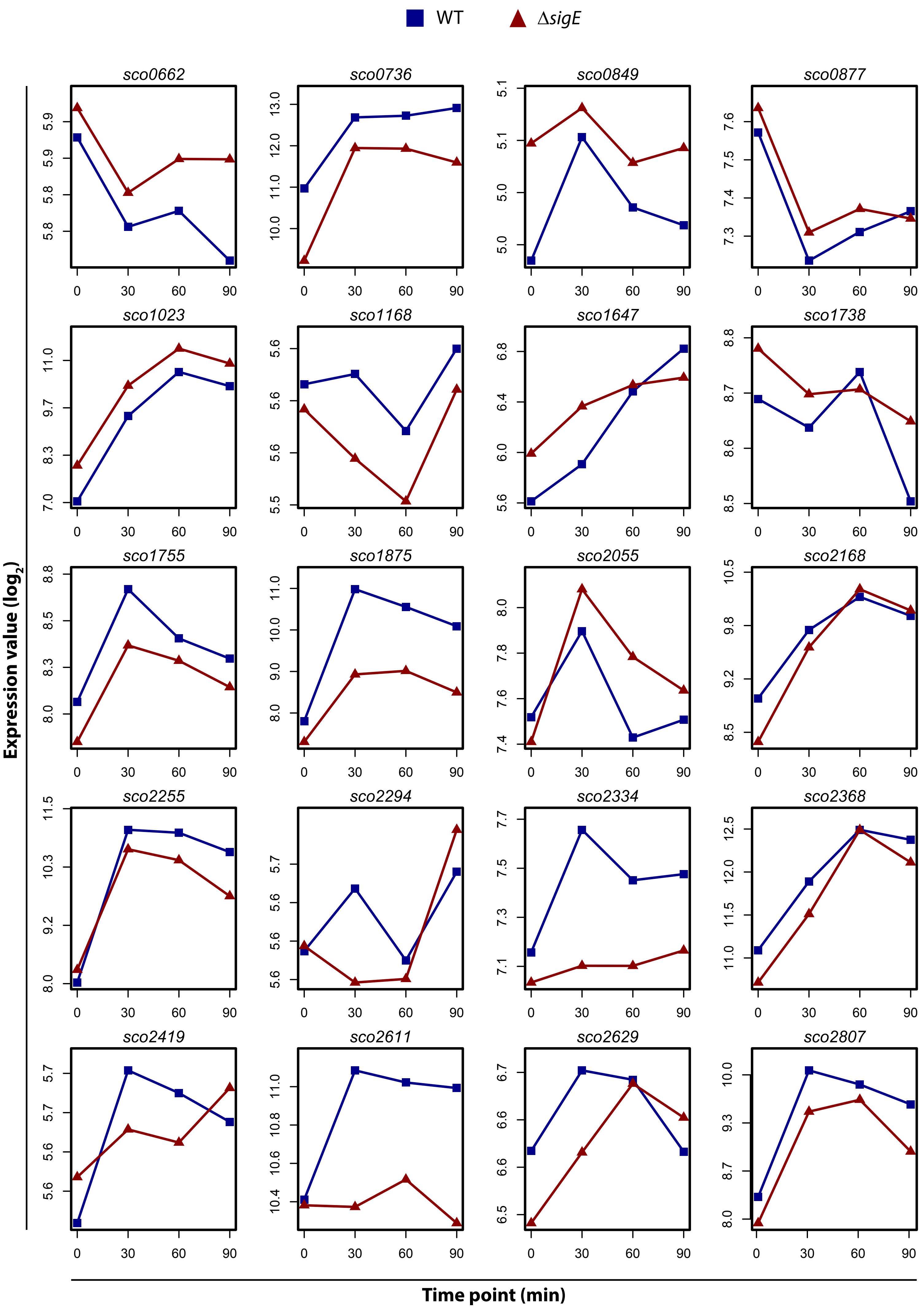

WT
ΔsigE
sco0662
sco0736
sco0849
sco0877
sco1023
sco1168
sco1647
sco1738
sco1755
sco1875
sco2055
sco2168
sco2255
sco2294
sco2334
sco2368
sco2419
sco2611
sco2629
sco2807
Expression value (log_2)
Time point (min)

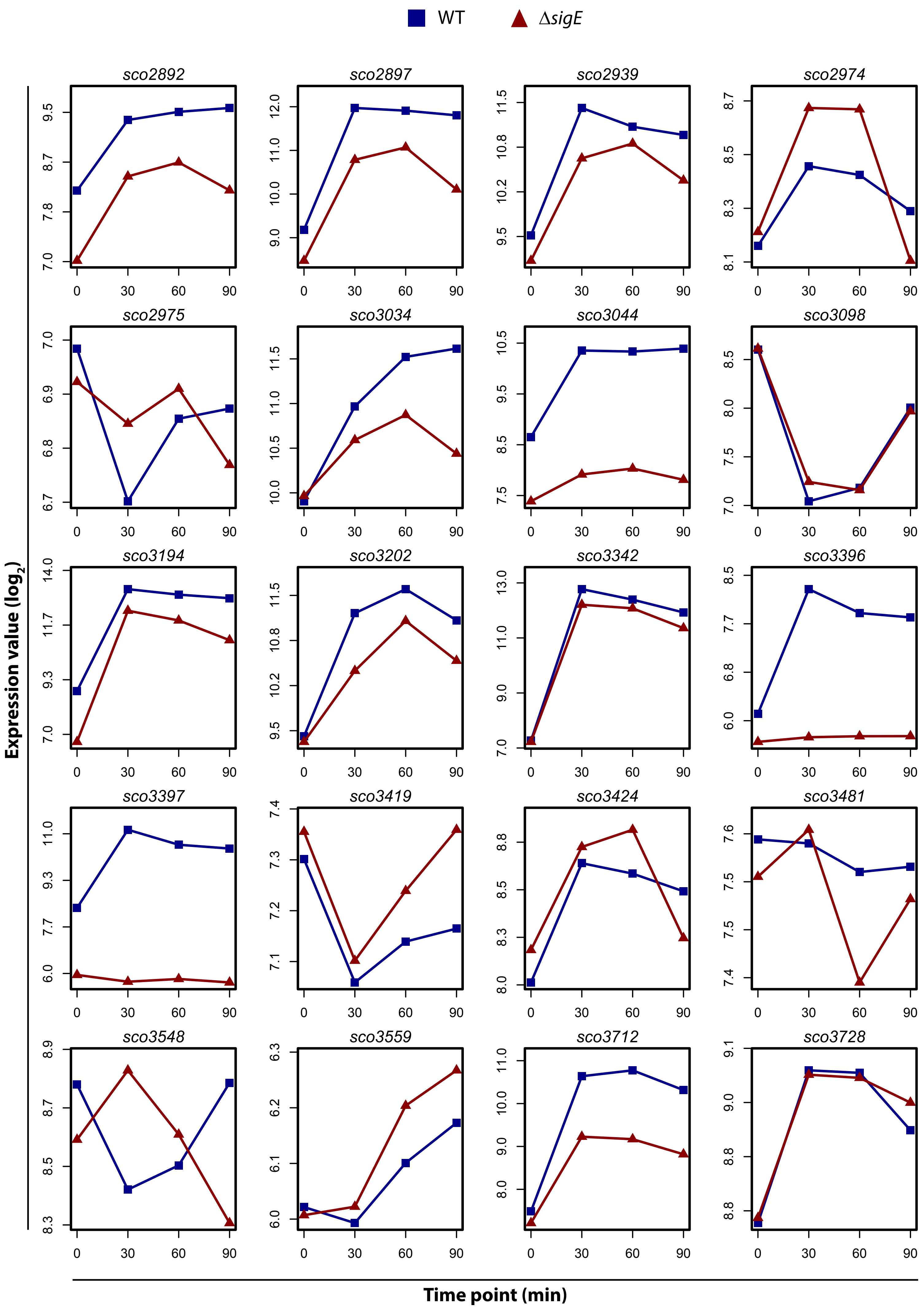

WT
ΔsigE
sco2892
sco2897
sco2939
sco2974
sco2975
sco3034
sco3044
sco3098
sco3194
sco3202
sco3342
sco3396
sco3397
sco3419
sco3424
sco3481
sco3548
sco3559
sco3712
sco3728
Expression value (log2)
Time point (min)

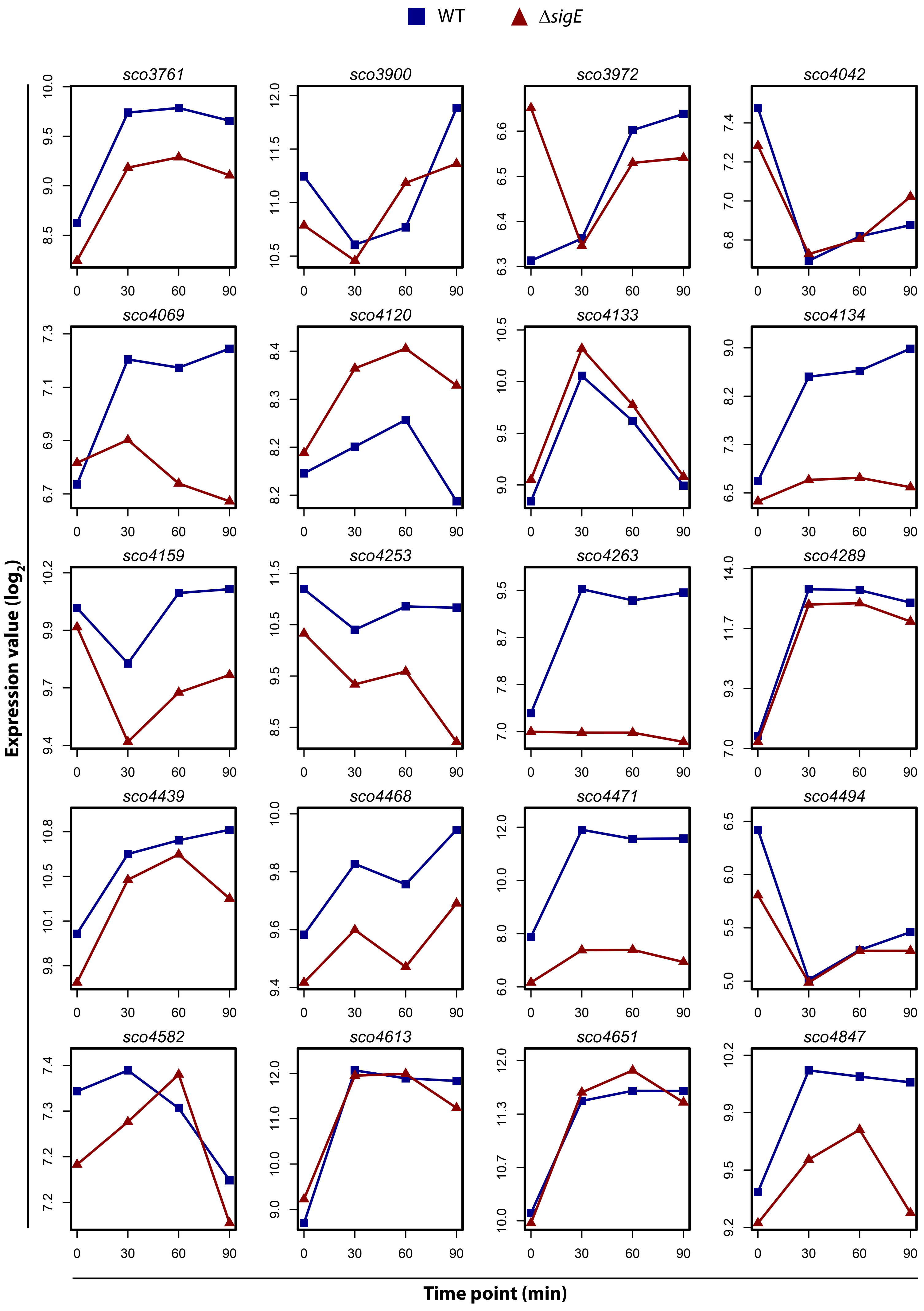

WT
ΔsigE
sco3761
sco3900
sco3972
sco4042
sco4069
sco4120
sco4133
sco4134
sco4159
sco4253
sco4263
sco4289
sco4439
sco4468
sco4471
sco4494
sco4582
sco4613
sco4651
sco4847
Expression value (log2)
Time point (min)
0
30
60
90

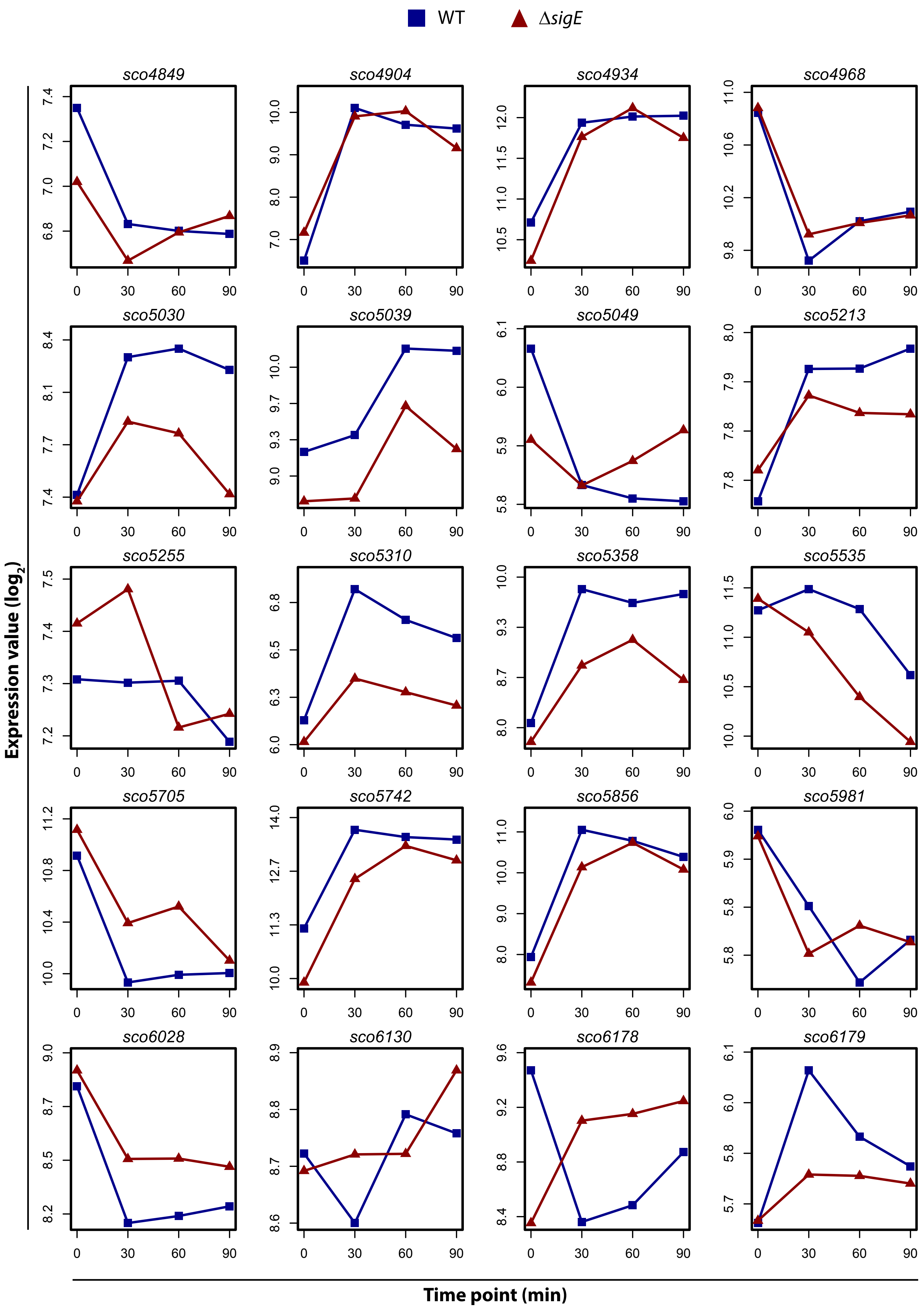

WT
ΔsigE
sco4849
sco4904
sco4934
sco4968
sco5030
sco5039
sco5049
sco5213
sco5255
sco5310
sco5358
sco5535
sco5705
sco5742
sco5856
sco5981
sco6028
sco6130
sco6178
sco6179
Expression value (log2)
Time point (min)

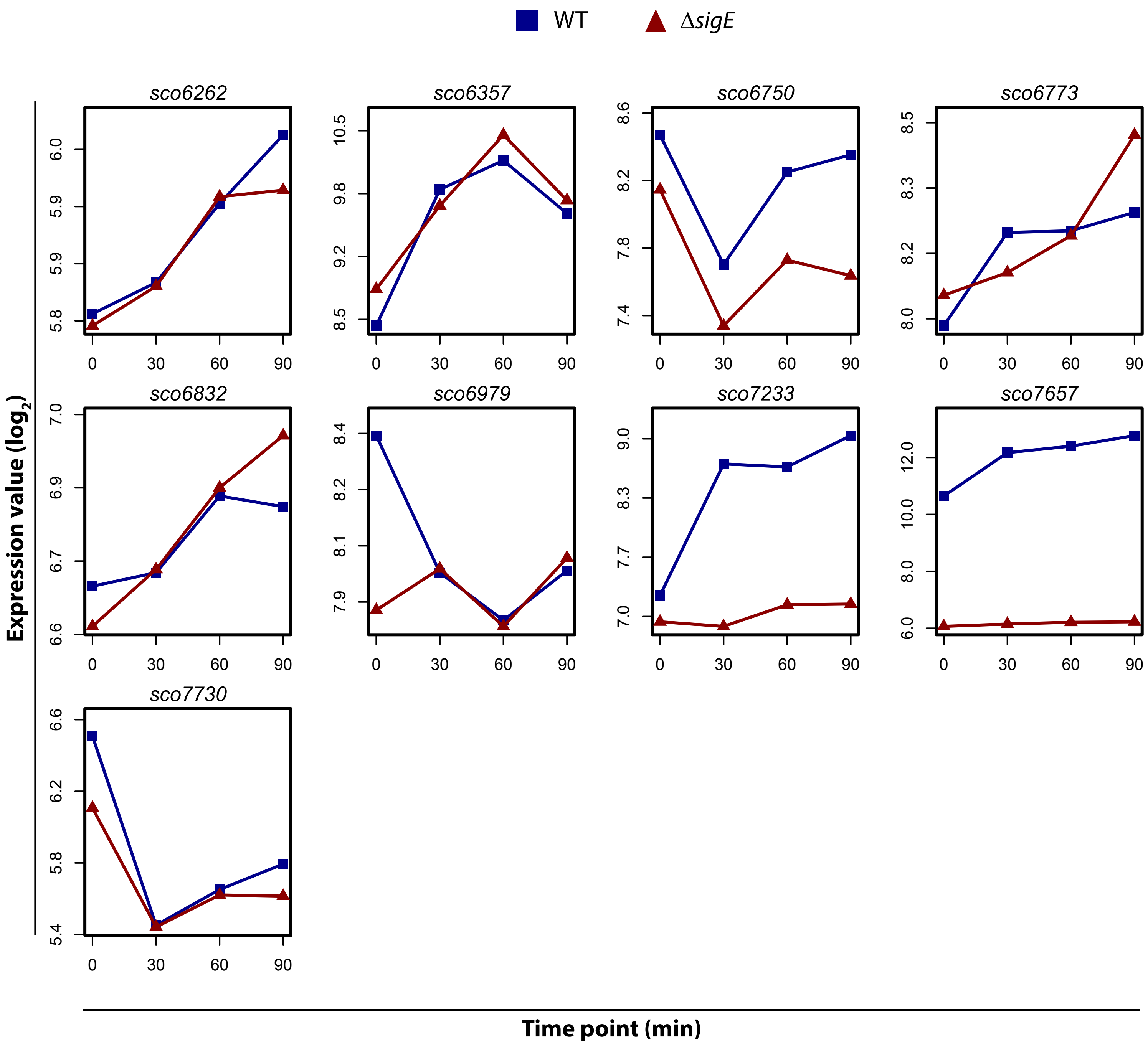

Fig. S2. Time-resolved microarray transcriptional profiling data for all the σ^E target genes listed in Table 1. Color-coding of the microarray data is as follows: *S. coelicolor* M600 (WT, blue squares), *sigE* null mutant J2130 (Δ*sigE*, red triangles). In each panel the x-axis indicates the time in minutes (0, 30, 60 or 90) after the addition of 10 µg/ml vancomycin, and the y-axis indicates the per gene normalized transcript abundance ($\log_2$). Note that two of the σ^E target genes listed in Table 1, the tRNA *scot11* and *sco6379*, were not represented on the Affymetrix Gene Chip and so no transcriptional profiling data are available for these two genes.

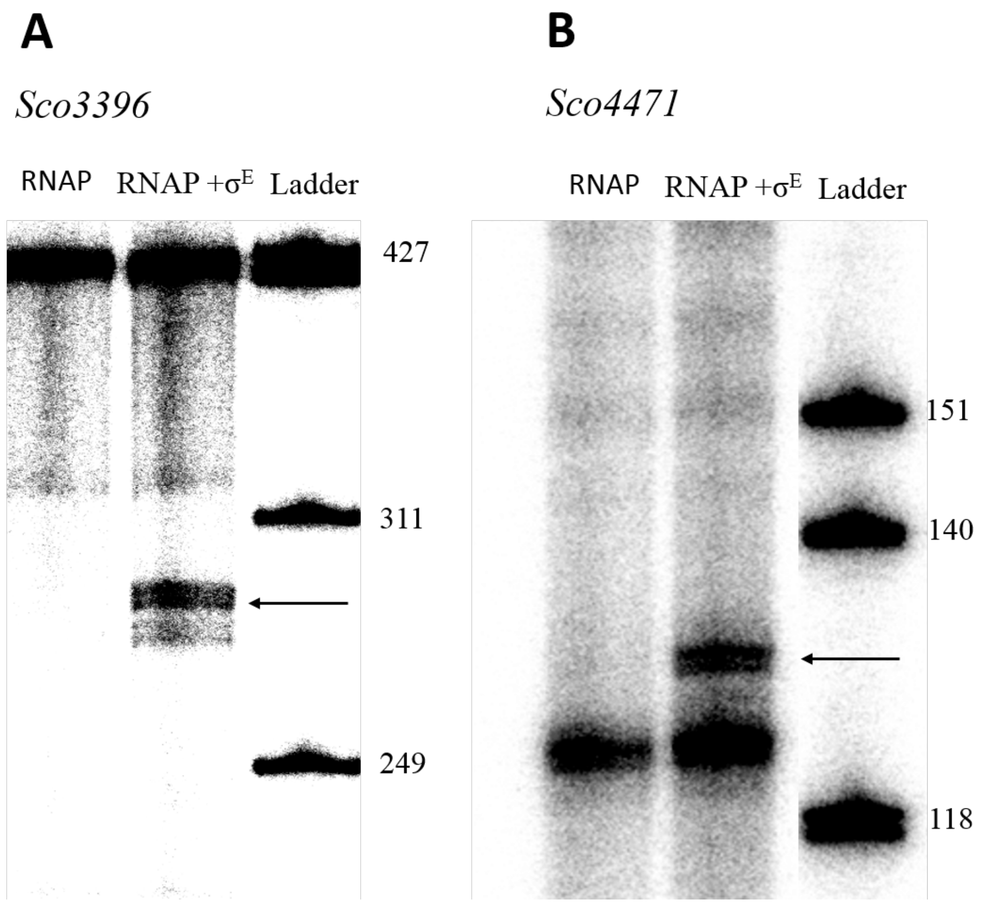

Fig. S3. *in vitro* run-off transcription assays on the σ^E target genes (A) *sco3396* and (B) *sco4471*. In each case a transcript of the expected size (indicated by the arrow) is only detected in the presence of RNAP and σ^E. The sizes of the standards (a heat-denatured ^{32}P-labelled *Hpa*II digest of pBR322) are indicated in nt.

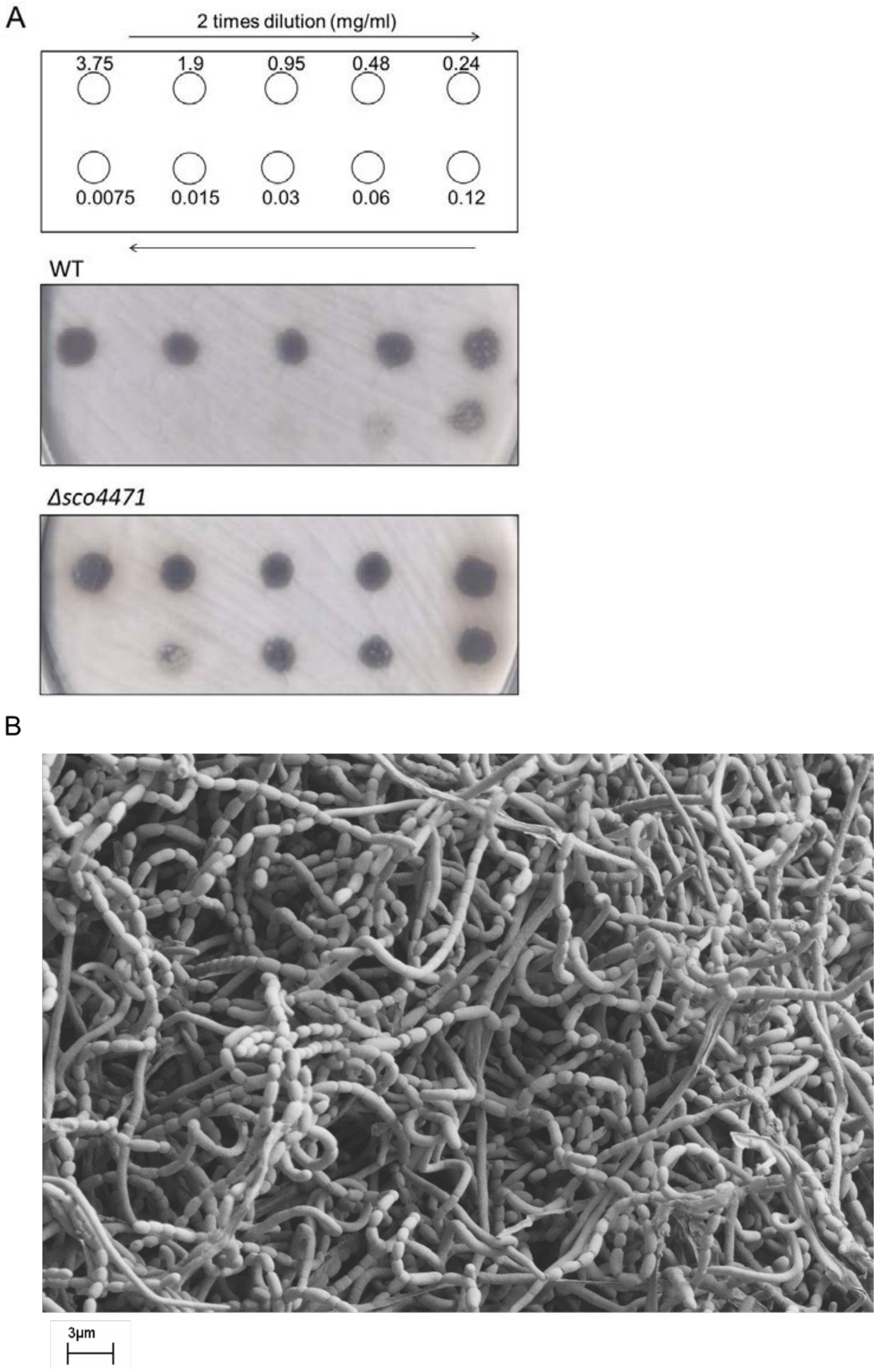

Fig. S4. (A) Lysozyme sensitivity of *Δsco4471::apr* mutant and the wild type *S. coelicolor* M600 (WT). A 2-fold dilution series of lysozyme was spotted onto DNA agar immediately after plating 2×10^6 spores of each strain to create a confluent lawn. Images were taken after 2 days of growth at 30°C. (B) Scanning electron microscopy image of the Δ*sco4471::apr* mutant showing minor abnormalities in spore size and shape. Imaged after 5 days of growth on SFM medium.

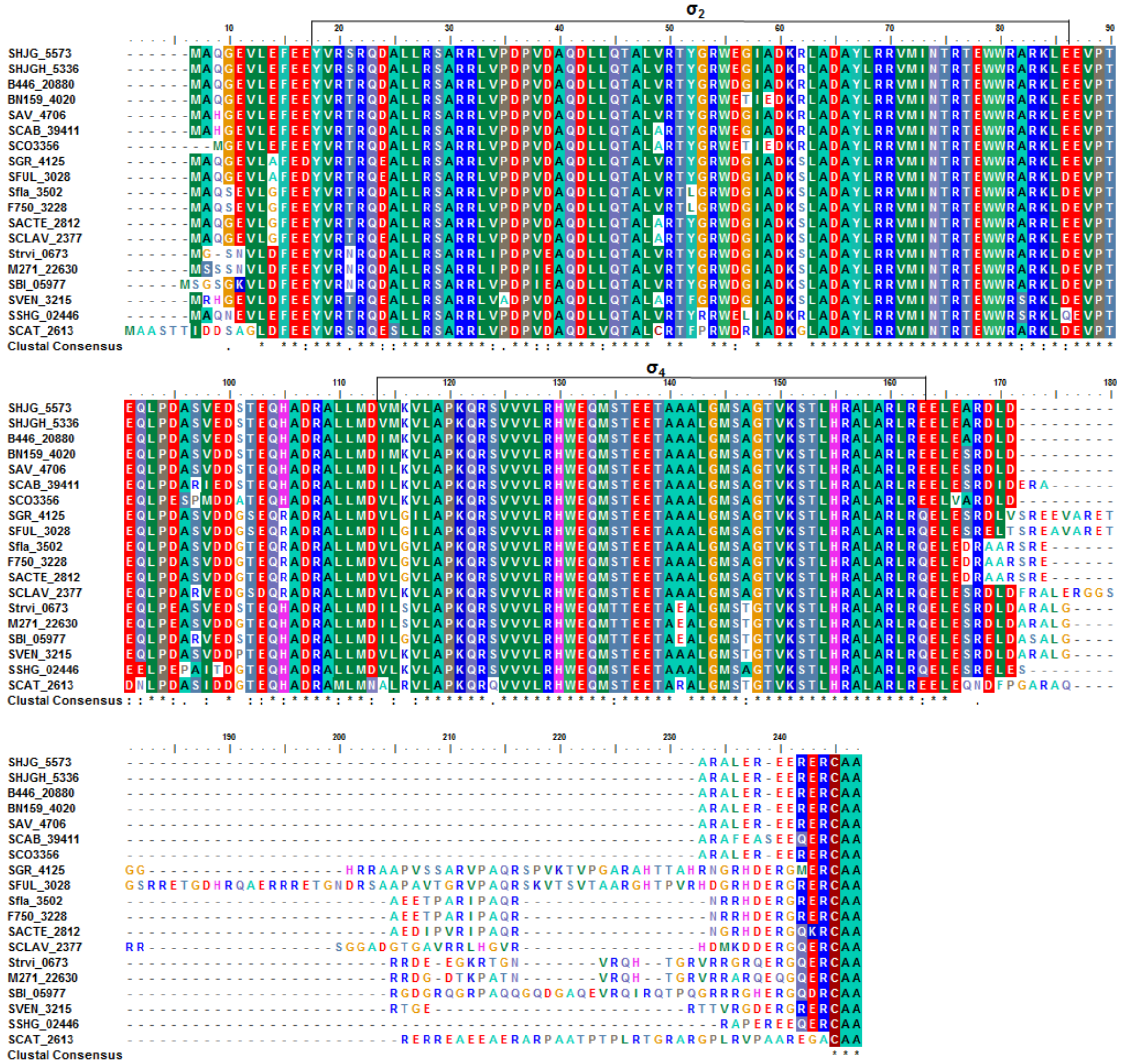

Fig. S5. Conservation of σ^E orthologs across 19 *Streptomyces* genomes. Multiple alignment of the amino acid sequences of the σ^E orthologs from 19 *Streptomyces* species (as listed in Table S2) were carried out by EMBL-EBI ClustalW2 online program (http://www.ebi.ac.uk/Tools/msa/clustalw2/). SCO3356 is *S. coelicolor* σ^E. The σ_2 and σ_4 domains were identified using the SMART database (http://smart.embl-heidelberg.de/) and are indicated in the figure.

Table S2. The 19 *Streptomyces* genomes used to analyze conservation of *S. coelicolor* σ^E target promoters

Accession[1]	Species	Abbreviation
CM000913	*Streptomyces clavuligerus* ATCC 27064	*Scla*
NC_003155	*Streptomyces avermitilis* MA-4680 = NBRC 14893	*Save*
NC_003888	*Streptomyces coelicolor* A3(2)	*Scoe*
NC_010572	*Streptomyces griseus* subsp. griseus NBRC 13350	*Sgri*
NC_013929	*Streptomyces scabiei* 87.22	*Ssca*
NC_015953	*Streptomyces sp.* SirexAA-E	*SspS*
NC_015957	*Streptomyces violaceusniger* Tu 4113	*Svio*
NC_016111	*Streptomyces cattleya* NRRL 8057 = DSM 46488	*Scat*
NC_016114	*Streptomyces pratensis* ATCC 33331	*Spra*
NC_016582	*Streptomyces bingchenggensis* BCW-1	*Sbin*
NC_017765	*Streptomyces hygroscopicus* subsp. jinggangensis 5008	*Shyg_1*
NC_018750	*Streptomyces venezuelae* ATCC 10712	*Sven*
NC_020504	*Streptomyces davawensis* JCM 4913	*Sdav*
NC_020895	*Streptomyces hygroscopicus* subsp. jinggangensis TL01	*Shyg_2*
NC_020990	*Streptomyces albus* J1074	*Salb*
NC_021055	*Streptomyces sp.* PAMC26508	*SspP*
NC_021177	*Streptomyces fulvissimus* DSM 40593	*Sful*
NC_021985	*Streptomyces collinus* Tu 365	*Scol*
NC_022785	*Streptomyces rapamycinicus* NRRL 5491	*Srap*

[1]The Genbank accession number (http://www.ncbi.nlm.nih.gov/genbank/) for the 19 *Streptomyces* genomes.

Table S3. Bacterial strains, plasmids and oligonucleotides.

	Relevant genotype/comments	Source/reference
S.coelicolor		
M600	SCP1$^-$ SCP2$^-$	(1)
J2130	*ΔsigE* M600	(2)
J3354	*ΔSCO4471::apr* M600	This work
E. coli		
DH5α	F' *supE44* Δ*lacU169* (Φ*80lacZ* Δ*M15*) *hsdR17 recA1 endA1 gyrA96 thi-1 relA1*	(3)
ET12567	F' *dam13::Tn9 dcm6 hsdM hsdR recF143::Tn10 galK2 galT22 ara-14 lacY1 xyl-5 leuB6 thi-1 tonA31 rpsL hisG4 tsx-78 mtl-1 glnV44*	(4)
ET12567 /pUZ8002	ET12567 containing helper plasmid pUZ8002	(2)
BW25113	*lacI*^{q} *rrnB*$_{T14}$ Δ*lacZ*$_{WJ16}$ *hsdR514* Δ*araBAD*$_{AH33}$ Δ*rhaBAD*$_{LD78}$	(5)
BL21 (DE3) /pLysS	F$^-$ *ompT gal dcm lon hsd*S$_B$ ($r_B^-m_B^-$) λ (DE3 [*lacI lacUV5*-T7 gene 1 *ind1 sam7 nin5*]) pLysS (Cam^R)	(6)
**Plasmid**		
pMS82	Plasmid integrating at the ΦBT1 attachment site (Hyg^R) of *S. coelicolor*	(7)
pIJ773	Plasmid template used for the amplification of the *apr oriT* cassette in 'Redirect' PCR-targeting	(8)
pIJ790	Modified λ RED recombination plasmid [*oriR101*] [*repA101*(ts)] *araBp-gam-be-exo*	(8)
pMS82-3FLAG-*sigE*	pMS82 harboring 3×FLAG-*sigE* driven by its native promoter	This work
**Primer**	**Primer sequences (5'-3')**	
SCO3396S1FW	CGATGTCCAGCAGGCCGAC	
SCO3396S1RV	GGTGGCGAGCAGCAGACCG	
SCO3397S1FW	CCCAGAACGGCCCGCACACTCCG	
SCO3397S1RV	GACGAGCACGCAGGCGCGGCCGAC	
SCO2897S1FW	CTGCCACGGAATTTCACGGGTG	
SCO2897S1RV	TAGACGTTGGACTGCTTGGTG	
SCO4134S1FW	CCACGTAGCCGCAGGCAATC	
SCO4134S1RV	AGGTGAGTGCGGTGCGCGTTC	
SCO3194S1FW	TGGTGACGCGGGCGATCGTGC	
SCO3194S1RV	GCCGCAGGCGGTGAGGGTGAG	
SCO4471S1FW	CTCCGTCGGCTACACCGAGCCC	
SCO4471S1RV	GAACCGGTGCCGCAGCCCGCGAG	
SCO5030S1FW	GGCAAGTACGCCTCCGCGGGC	
SCO5030S1RV	TTCCTCGCGGCGCTGCTCCAAC	
SCO7233S1FW	TCCAGCGACGACTGCCCGTTC	
SCO7233S1RV	TACGGATGCCGGGCCGCCTG	
SCO2334S1FW	CGATGGAACCCTTCCTCGCAAAG	
SCO2334S1RV	CGAGCAGCGGCACGCGATG	
SCO2611S1FW	GTCTGATCCTTCTTCGGGA	
SCO2611S1RV	TTGGCGGTCCCGAGGTCGAC	
SCO5358S1FW	CACCTACGCCTCCCACCTG	
SCO5358S1RV	CGCGACGAGCAGGGCCTTG	
SCO4934S1FW	AGTGGGACCTCGACGAGGATG	
SCO4934S1RV	GGAACATGCCGAGAGCACCAG	

SCO3712S1FW	ACCGTCGTCTACACGGCCG
SCO3712S1RV	ACCACCGTGCCGCGCCATG
SCO4263S1FW	TCCGGAGCGGACACCGCCAC
SCO4263S1RV	GCAGGACGAGGCGGCCGCTG
SCO3044S1FW	TCGCGCGTTTGTCGAGTGCGCGAAGTGACCT
SCO3044S1RV	GCTGGTAGTAGAGGTATCCGG
SCO7657S1FW	CAACACCTTGCACGTGGTCACC
SCO7657S1RV	GAGTCCGGCGGTCACGGCCATG
SCO4847S1FW	CGTCGTATCCCCTCGGTTG
SCO4847S1RV	ACGGACTCGTCGACGGGCTC
SCO4471KOFW	CCAGTGACCACCAAGGACGCGACGAACGAG
	GTTGCGATGATTCCGGGGATCCGTCGACC
SCO4471KORV	GGAAACCCGCATTTCAGGCACCGTTTCGCGG
	TCGCTTCATGTAGGCTGGAGCTGCTTC
$P1_{3NFLAG\text{-}SigE}$	AAAAAAGCTTACCAGGTGTGGCAGGAGC
$P2_{3NFLAG\text{-}SigE}$	TCGATGTCGTGGTCCTTGTAGTCGCCGTCGTG
	GTCCTTGTAGTCCATGTCAACCGCCTTCCG
$P3_{3NFLAG\text{-}SigE}$	CGACTACAAGGACCACGACATCGACTACAAG
	GACGATGACGACAAG
	GGCGAGGTGCTCGAGTTC
$P4_{3NFLAG\text{-}SigE}$	AAAAGGTACCCACCGCCGTACTCGCC

References

1. Chakraburtty, R., and Bibb, M.J. **(**1997) The ppGpp synthetase gene (*relA*) of *Streptomyces coelicolor* A3(2) plays a conditional role in antibiotic production and morphological differentiation. J. Bacteriol. **179:** 5854-5861.
2. Paget, M.S., Chamberlin, L,, Atrih, A., Foster, S.J., and Buttner, M.J. **(**1999) Evidence that the extracytoplasmic function sigma factor σ^E is required for normal cell wall structure in *Streptomyces coelicolor* A3(2). *J Bacteriol* **181:** 204-211.
3. Hanahan D. 1983. Studies on transformation of *Escherichia coli* with plasmids. J Mol Biol **166:** 557-580.
4. MacNeil, D.J., Gewain, K.M., Ruby, C.L., Dezeny, G., Gibbons, P.H., and MacNeil, T. **(**1992) Analysis of *Streptomyces avermitilis* genes required for avermectin biosynthesis utilizing a novel integration vector. *Gene* **111:** 61-68.
5. Datsenko, K.A., and Wanner, B.L. **(**2000) One-step inactivation of chromosomal genes in *Escherichia coli* K-12 using PCR products. *Proc Natl Acad Sci USA* **97:** 6640-6645.
6. Studier, F.W., and Moffatt, B.A. **(**1986) Use of bacteriophage T7 RNA polymerase to direct selective high-level expression of cloned genes. *J Mol Biol* **189:** 113-130.
7. Gregory, M.A., Till, R., and Smith, M.C. **(**2003) Integration site for *Streptomyces* phage *φBT1* and development of site-specific integrating vectors. *J Bacteriol* **185:** 5320-5323.
8. Gust, B., Challis, G.L., Fowler, K., Kieser, T., and Chater, K.F. **(**2003) PCR-targeted *Streptomyces* gene replacement identifies a protein domain needed for biosynthesis of the sesquiterpene soil odor geosmin. *Proc Natl Acad Sci USA* **100:** 1541-1546.
